# The Baobab (*Adansonia digitata* L.) in Southern Kenya–A Study on Status, Distribution, Use and Importance in Taita–Taveta County

**DOI:** 10.1007/s00267-020-01311-7

**Published:** 2020-06-12

**Authors:** Sahrah Fischer, Lisa Jäckering, Katja Kehlenbeck

**Affiliations:** 1 Institute of Agricultural Sciences in the Tropics (Hans-Ruthenberg-Institute), University of Hohenheim, Garbenstr. 13, 70599 Stuttgart, Germany; 2 International Fund for Agricultural Development (IFAD), Via Paolo di Dono, 44 00142 Rome, Italy; 3 World Agroforestry Centre (ICRAF), United Nations Avenue, Gigiri PO Box 30677, Nairobi, 00100 Kenya; 4 Faculty of Life Sciences, Rhine-Waal University of Applied Sciences, Marie-Curie-Straße 1, 47533 Kleve, Germany

**Keywords:** Regeneration, Non-timber forest products (NTFPs), Multipurpose tree, Land use, Conservation

## Abstract

Baobab (*Adansonia digitata* L.) is a multipurpose, drought resistant, wild fruit tree, endemic to arid and semi-arid lands of Sub-Saharan Africa. Baobab populations have been showing a lack of regeneration, and therefore causes concern for the species survival. This study investigated the state, distribution and use of baobabs in an under-researched population in Kenya, to identify the potential for further use and development of baobab resources. A baobab population was chosen in Taita–Taveta County, covering a sample area of 2015 km^2^. A systematic stratified transect survey was done to map baobab distribution using 49 transects (0.5 × 3 km each). The diameter at breast height and other indicators were measured on all baobabs in the transects to assess population status and health. A household survey (*n* = 46) and focus group discussions (*n* = 12) were done following the transect survey to gain an idea on the uses and distribution of baobab. In total, 432 baobab trees were measured and recorded in the research area of 2015 km^2^. The baobabs grew in two clusters (i.e., areas with a baobab density of ≥0.08 baobabs/ha). Both clusters showed rejuvenating populations. The main factors identified by the respondents, positively and negatively influencing baobab distribution were environmental factors, wildlife, human impact and commercial value. The study area shows a great potential for baobab to become an important part of the diet, due to its current use as an emergency food during food scarce times, and the relatively healthy and stable rejuvenating populations.

## Introduction

Baobab (*Adansonia digitata* L.) is a multipurpose, drought resistant, wild fruit tree, that is often found in arid and semi-arid lands (such as savannahs) of Sub-Saharan Africa (SSA) (Sidibe and Williams 2002a, b; Wickens and Lowe 2008). As a multipurpose tree, almost all parts of the tree are used. While the leaves and the fruits of the baobab provide important and nutrient-rich food sources, different tree parts are also used for medicine, handicraft, shelter, fertilizer and fodder (Gebauer et al. 2002). Baobab often has a cultural or religious value. It also provides habitats for many wild animals, as well as other ecosystem services such as carbon sequestration, soil enrichment, air and water quality improvement and biodiversity conservation (Wickens and Lowe 2008; Gebauer and Luedeling 2013). The valuing of baobab food products for subsistence and income generation varies in local communities throughout Africa. In some areas such as Sudan or Mali, the baobab is highly valued for its food production, drought resistant properties and medicine production. In other areas such as South Africa, people have substituted some baobab products (while retaining other such as shading and consuming the fruits and seeds), and do not value the tree as highly anymore (Venter and Witkowski 2013a, b). It is, however, still used as a coping strategy during food scarce times as it bears fruits during the dry season and the pre-harvest time (Gebauer et al. 2002).

The high concentration of nutrients in the fruit pulp (mainly vitamin C and other micronutrients as well as antioxidants) has been recognized on an international level, triggering export from mainly western and southern Africa to the European Union (EU) and United States of America (USA). Baobab fruit pulp is marketed as a ‘superfood’ (Buchmann et al. 2010; Cuni-Sanchez et al. 2010; Sidibe and Williams 2002a, b; Wickens 1982; Gebauer et al. 2013). Therefore, the baobab and its products are not only important for food and nutrition security, but could also provide a source of income for resource-poor farmers, particularly in the drylands of SSA (Jäckering et al. 2019).

The demand for healthy foods, or ‘superfoods’ such as the baobab, is increasing worldwide. Africa has also seen an increase in this demand, and therefore the baobab has the potential to be, and in some countries has already become, an important healthy food (Buchmann et al. 2010). The increasing popularity of baobab in the EU and USA has triggered concern for the baobab populations. In countries such as South Africa, Malawi and Burkina Faso, with areas containing large baobab populations, scientists have found a lack of juvenile baobabs leading to potentially decreasing stands (Wickens and Lowe 2008; Venter and Witkowski 2013a, b). The baobab is a wild tree and has not been domesticated, nor was it being planted in the study area and most of the other countries of SSA. Therefore, all resources collected from the baobabs come from natural regeneration in these areas. Some uses of baobab products may have negative effects on the tree such as high levels of leaf harvest, which can reduce fruit production (Dhillion and Gustad 2004). An increased level of fruit harvest to satisfy both local and international fruit pulp demand results in enhanced removal of seed material and may endanger the rejuvenation of current baobab stands (Cuni-Sanchez 2010). Higher land use intensities have been observed to cause habitat loss for baobab trees (Birhane et al. 2020). In Burkina Faso, land use has also been observed to affect baobab populations, as the national parks (NP) showed a higher regeneration of baobab trees than cropland or fallows (Schumann et al. 2010). In South Africa, higher baobab densities, but lower recruitment rates were found in villages and fields as compared with plains and rock outcrops (Venter and Witkowski 2010).

In Kenya, there is some information available on the general distribution of the species (Maundu 1999). However, little is known about tree densities, stand structure and population health, possible threats to the genetic resources and the level of importance of the trees to local communities in Kenya (Omondi et al. 2019). The main objective of this study was therefore to gather information on structure, use and importance of an undocumented baobab population in southern Kenya, in a diverse landscape. Specifically, the study aims to answer three questions: (1) what effect does land use have on the baobab population? (2) Is the baobab population threatened by a lack of rejuvenation, overutilization, pests or diseases? (3) How does the local community perceive the baobab tree and how do human activities such as farming or building affect the baobab population? As the research area provides the opportunity of division into different land use intensities, we hypothesize that the lowest land use intensity (such as a NP) would contain the highest density of baobabs, due to the low level of human disturbance. On the other hand, the highest land use intensity (such as large-scale single crop plantations (e.g., sisal)) would have the lowest density of baobabs due to the high level of disturbance such as high intensification and mechanization.

## Materials and Methods

### Research Area

**Fig. 1:**
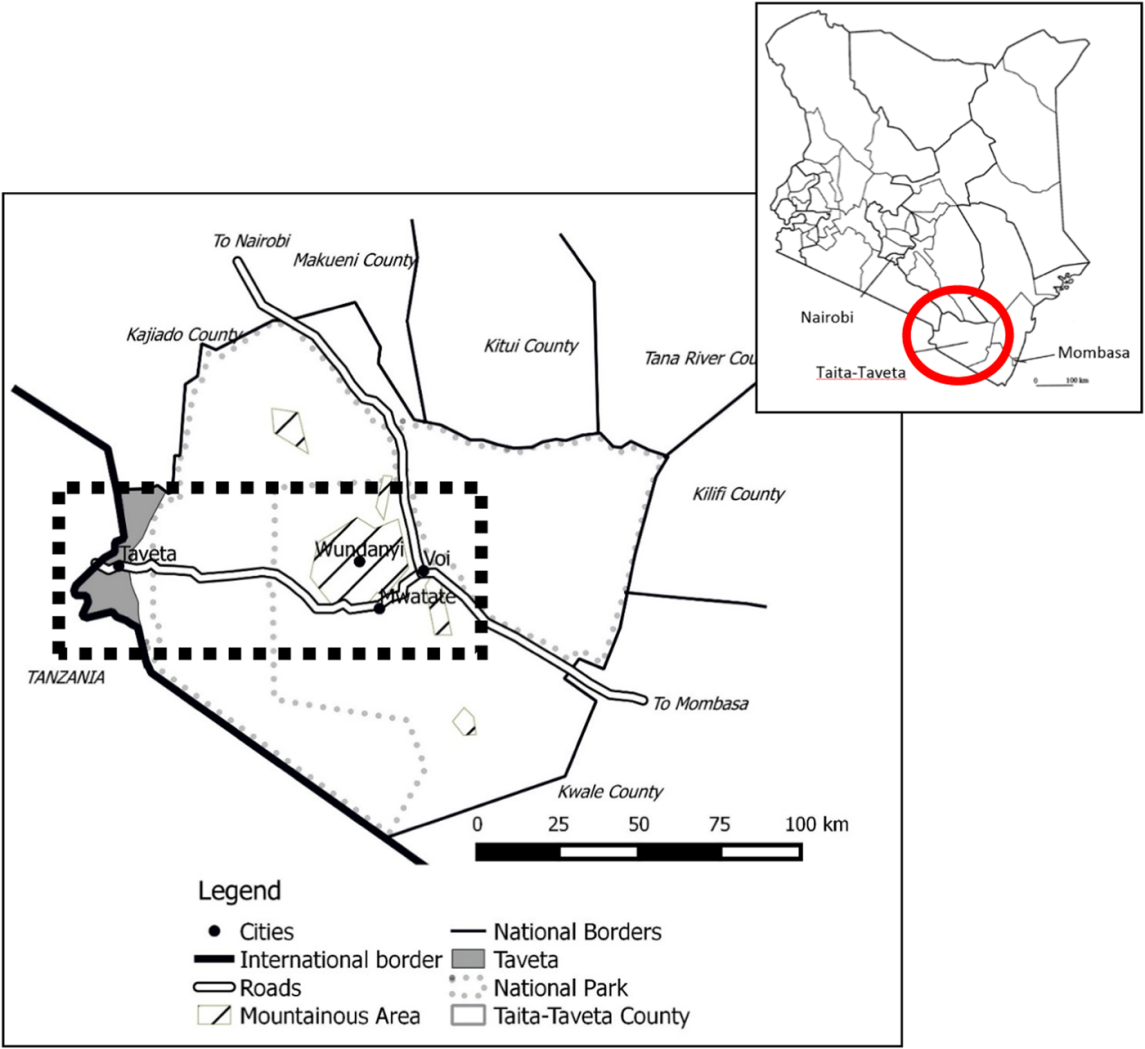
Map of Taita–Taveta County, Kenya. Image made using QGIS Brighton. Adapted from: Dijkstra and Magori (1994) and MEMR (2009). Area in the rectangle is the sample area, selected in such a way as to cover all topographic formations in the region

The study was performed in Taita–Taveta County covering an area between the towns of Voi and Taveta (Fig. 1), with a documented baobab population (Maundu 1999). The number, state and distribution of baobabs, however, were unknown as was knowledge on the use levels and importance of baobab for livelihoods of local communities.

Taita–Taveta has a semi-arid climate, characterized by two rainy seasons, one from March to May/June and the second from October to December. The average annual rainfall varies between 500 mm in the plains and 1500 mm in the hills (MEMR 2009; Pellikka et al. 2004). Agro-ecological zones covered in this study in Taita–Taveta range from the upper midland zones (1220–1680 m asl), lower midland zones (790–1220 m asl), to the lowland zones (L, <790 m asl) (Jaetzold and Schmidt 1983; Dijkstra and Magori 1994). Soil types range from cambisols on the hills, to luvisols and arenosols on the footslopes and acrisols and ferralsols in the lower lands, interspersed with solonetz and fluvisols (Jaetzold and Schmidt 1983). The natural vegetation varies from Somalia–Masai–Acacia bushland and thicket and Afromontane undifferentiated forest (Kindt et al. 2015; van Breugel et al. 2015a, b). In Taita–Taveta, about 62% of the total area is NP, 24% is rangeland, 12% of the area is agricultural land (mainly rainfed) and 3% is rocky and watery areas (Dijkstra and Magori 1994). The main large-scale farming present in the research areas was sisal (*Agave sisalana*) production, with two remaining working sisal estates, the plantations Taita and Voi (MEMR 2009), and one inactive plantation in Taveta. Land use varies from intensive agriculture in the highlands, to extensive agriculture and livestock grazing in the lowlands.

### Research Design

**Fig. 2:**
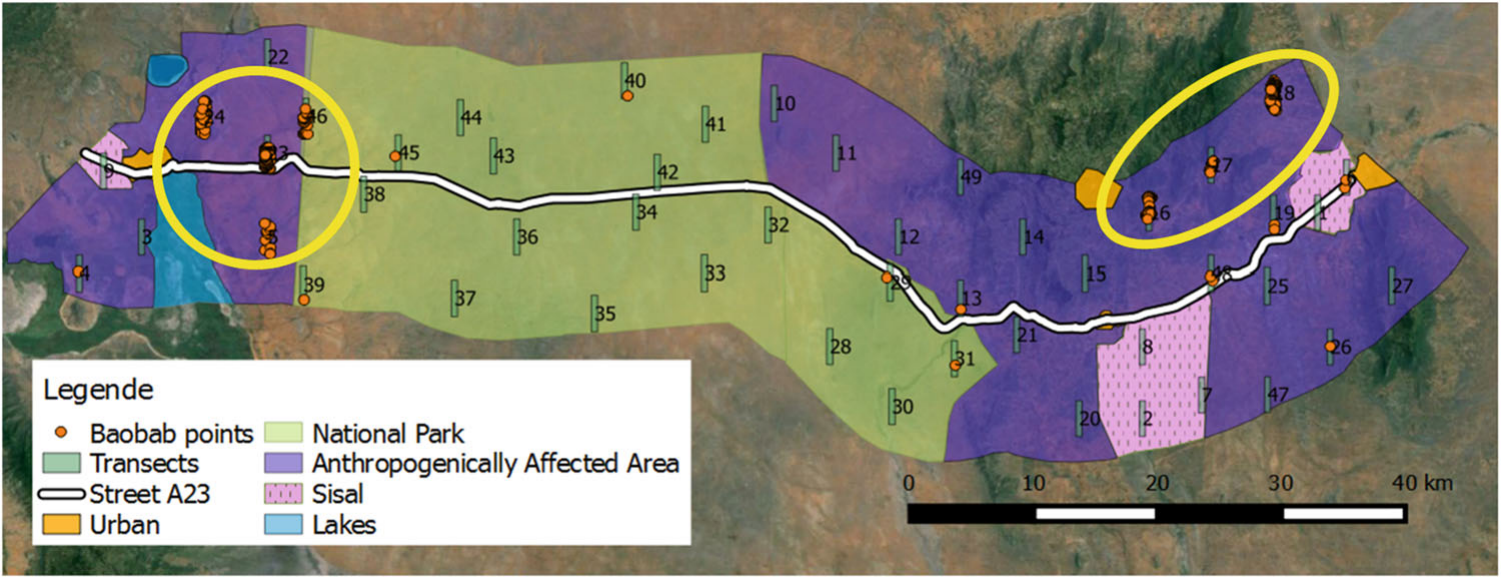
Map showing the locations of the baobab trees (orange points) found in the 49 surveyed transects along the Voi–Taveta road in Taita–Taveta County, Kenya. Each yellow circle marks areas of high baobab density (transects with ≥0.08 baobabs/ha); markings were based on transect counts. The circle on the right is named the ‘Taita cluster’, whereas the one on the left is named ‘Taveta cluster’. Map made on QGIS Valmiera 2.2 with a GoogleEarth background

A mixed methods approach was used, combining quantitative and qualitative data sets (Johnson et al. 2007). In a mixed method approach, the quantitative and qualitative data sets support each other. In this case, particularly, the qualitative information was used to supplement and complete the data collected quantitatively. The sample area was constructed around the main road (A23) leading from Voi to Taveta (Fig. 1). This road was selected not only to facilitate access to the research plots and respondents, but also because it cuts through different land use systems with different intensities of human disturbance. QGIS Valmiera 2.3.9 was used to construct a 20 km buffer around the A23 Voi–Taveta road resulting in a sample area surrounding the road, with a total area of 2015 km^2^ (Fig. 2). The borders of the sample area were, on the Taveta side at the border to Tanzania, and on the Voi side at the Nairobi–Mombasa highway. The sample area was stratified into three main land use types to identify any effects of land use on the baobab population. The first stratum was the ‘National Park’ (NP) with a total size of 789 km^2^, which was part of the Tsavo West NP, and represented the hypothesized lowest level of disturbance. The second stratum was the ‘Anthropogenically Affected Area’ (AAA) with a total size of 1125 km^2^ and included the area where the local population lived and practiced their income generating activities such as farming, representing the hypothesized medium level of disturbance. It also included savannah or wooded areas, accessed by the local population. The third stratum was Sisal plantations (Sisal) (size 101 km^2^), which consisted of intensive, highly mechanized farming, and was assumed to be a highly disturbed baobab habitat. The urban areas of Voi, Wundanyi, Mwatate and Taveta were excluded from the strata formation and from the entire survey, due to a lack of trees within these locations (Figs 1 and 2).

The baobab population was mapped using a systematic stratified transect survey. The transects were placed into the strata using a 5 × 5 km grid laid over the map perpendicular to the road at alternating heights to include any possible road gradient (Quinn and Keough 2002). To include a random component into the sampling method (Kindt and Coe 2005), the transects were placed at randomly selected heights in each grid box. The starting and end points of the transects were therefore defined by their placement in the grid. The starting and end points of the research area were from Voi town to the Kenyan/Tanzanian border. Systematic sampling was chosen to allow the description of baobab distribution along (a) an environmental gradient (distance from NP as well as the different topographic formations such as savannah plains, flat lake-side areas and hilly to mountainous areas); and (b) a road gradient from the highway (Quinn and Keough 2002). The transects had a size of 0.5 × 3 km, giving them an area of 1.5 km^2^ each. An initial number of 50 transects for the whole sample area was selected to cover a representative area in each stratum. However, one transect was dropped as it overlapped on the borders of two strata, resulting in 49 final transects. The number of transects per stratum was calculated and distributed according to the surface area of the stratum, resulting in 24 transects for AAA, 19 transects for NP and 6 for S (Fig. 2).

The transect coordinates were loaded onto a Garmin GPS 60 s, and located in the field using the GPS. The transects were covered on foot to increase the probability of detection of any baobab tree, with a team ranging from 3 to 5 people.

When encountering a baobab in a transect, different measurements were taken. These included taking the GPS coordinates (Garmin GPS 60 s), measuring tree height (m), noting the level of debarking based on the scale by Mpofu et al. (2012), denoting the presence of flowers, fruits or leaves, and measuring the circumference at breast height (CBH) at a height of 1.30 m to calculate the diameter at breast height (DBH) (please find more detailed information in the supplementary materials—Methods S1).

Debarking is dangerous for baobab trees as it exposes the inner soft wood to the environment. The three biggest ‘debarkers’ in the research area were found to be elephants (who gauge open the bark to chew on the soft wood for water during drought) (Mpofu et al. 2012), insects and humans (harvesting bark fibre, or for construction purposes). Their damage was recorded and assessed on a scale by Mpofu et al. 2012 (further information in the supplementary materials).

Individual household surveys were conducted in the area. The household surveys covered questions related to phenology, presence, use levels and importance of baobab to the local farmers. Forty-six farmers were interviewed within the 24 AAA transects. Two respondents were purposefully selected per transect. The household survey was held between the 29 May 2014 and 7 July 2014. Out of the 46 respondents, 26 women and 20 men between the ages of 19 and 79 were interviewed.

**Fig. 3:**
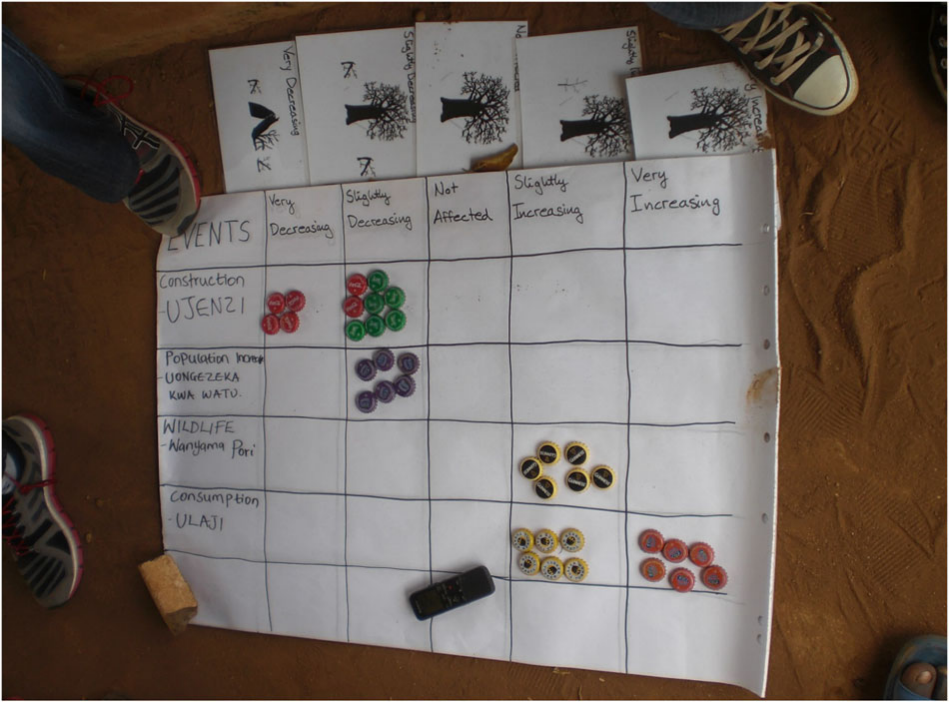
The occurrence game used in focus group discussions in Taita–Taveta, Kenya, to understand what events (selected by the participants) affected the baobab population based on the scale shown in the first row of the table (abundance of trees very decreasing, slightly decreasing, not affected, slightly increasing and very increasing). Bottle caps were used as game pieces for each participant to make their selection

In addition, 12 focus group discussions (FGDs) were conducted separately in low-baobab density areas (four FGD locations) and high baobab density areas (four FDG locations). The villages where the FGDs were conducted, were selected randomly per density area (low-density area and high-density area) (Fig. 5S). A total of eight participants were each selected through the respective village chief. As the aim of the FGDs was to understand any changes in baobab population over time, as well as traditions related to baobab, only village elders were invited. The groups were split by gender, resulting in a male FGD and a female FGD in each location. To begin, the respondents were asked a series of guiding questions on perceptions, use, importance and distribution of baobab in the area. Following this, factors affecting the baobab population were identified through an ‘occurrence game' (Catacutan et al. 2014). The game was set up as a grid with the top row featuring the effect on baobab trees (abundance of trees very decreasing, slightly decreasing, not affected, slightly increasing and very increasing). The first column was used to name events that affected the baobab population, and bottle caps were then placed into the corresponding cell by participants depending on the level of effect the event had on the baobab population (Fig. 3). Events affecting the baobab population were selected by the participants. In addition, topics such as use, importance and traditional beliefs about the baobab tree were discussed in the FGDs, but separately from the occurrence game. The FGD guideline can be found in the supplementary material, Methods, S3.

### Data Analysis

The baobab distribution was first analyzed using the predefined land use types. Subsequently, due to their distribution patterns, the baobabs were divided into high-density areas and low-density areas, to allow for a better analysis. The high-density areas contained transects having a baobab density ≥ 0.08 baobabs/ha, and low-density areas with a density of <0.08 baobabs/ha, which was the overall mean baobab density found in the research area.

From the CBH data collected from each of the baobab trees in the surveyed transects, the DBH was calculated. This was used to classify the baobabs into different size classes, and to estimate the count of baobab trees for each size class in the different land use areas (Gebauer and Luedeling 2013).

For the size class analysis and subsequent discussion, baobabs were separated into two categories: (1) large baobabs, having a DBH ≥ 1 m and (2) small baobabs with a DBH < 1 m (Venter and Witkowski 2010), or with a height of <1.30 m (where no DBH could be measured). Small baobabs included juveniles, sub-adults and ‘stunted’ baobabs (see description in the ‘Results’ section). The data was tested for normality using the Kolmogornov–Smirnoff test. The test revealed that the data was not normally distributed, and strongly positively skewed. All statistical tests were done using the SAS 6.0 statistical programme.

Size-class distribution (SCD) curves were calculated using the DBH, and used to analyse the age distribution. By plotting SCDs, the reproductive behaviour and age of the population stands can be compared. The SCDs were calculated after the methods of Condit et al. (1998) and Lykke (1998), and the slopes used as an indication of population structure (Lykke 1998). To calculate the SCDs, DBH classes were made and the number of trees per DBH class plotted as a bar graph. Curves were fitted to the bar graph to calculate the slopes for further analysis (Condit et al. 1998; Lykke 1998). The interpretation of the curves was based on Obiri et al. (2002). If the slope is negative, meaning that there are more elements in the smaller size classes, there is recruitment. Flat slopes of zero show an equal number of old and young trees. Flat SCDs can indicate a lack of rejuvenation or a declining population, but it can also be caused by very fast growth in smaller size classes or a higher survival rate (Lykke 1998; Cuni-Sanchez 2011b). Positive slopes signify no regeneration or episodic recruitment for the long-lived species (Obiri et al. 2002; Venter and Witkowski 2010). The resulting SCD values were compared on SAS 6.0 statistical programme using the PROC GLIMMIX procedure.

In order to understand the underlying reasons for the quantitatively measured baobab distribution, FGDs were conducted. The FGDs were first transcribed, then post-coded and combined with the household survey results. The post-coding used split respondent comments into separate categories such as, positive and negative views on baobab tree presence. The qualitative analysis was then split between the FGDs done in the low-density areas and high-density areas, as the context of different baobab population densities was bound to affect the answers given in the FGDs and the individual survey. A phenological calendar with regard to leaf emergence and fall, flower occurrence and fruit development and maturity seasons was produced as a result of the responses of the questionnaires. FGDs were then conducted to understand when the different phenological stages occur in comparison to rainy seasons and agricultural activities such as fruit harvesting. In addition, the events leading to baobab population change were analyzed after performing the occurrence game, by counting the bottle caps per field, and evaluating the importance and frequency of each event by the information given by the respondents. The types of use of baobab parts and the baobabs’ importance for local farmers were also evaluated and compared between the low-density areas and the high-density areas.

The discussion will be structured using the data of both the quantitative and the qualitative methods to discuss the distribution and regenerative behaviour of the baobabs. Since the two data sets complement each other, they will not be separately labelled, but used together in the discussion.

## Results

### Baobab Distribution in the Three Surveyed Land Use Systems

In total, 432 baobab trees were mapped in the surveyed 49 transects, covering a total area of 73.5 km^2^. Of these 432 trees, only 2 were found in the land use system Sisal, 34 in the NP and 396 in the AAA (Fig. 2).

**Fig. 4:**
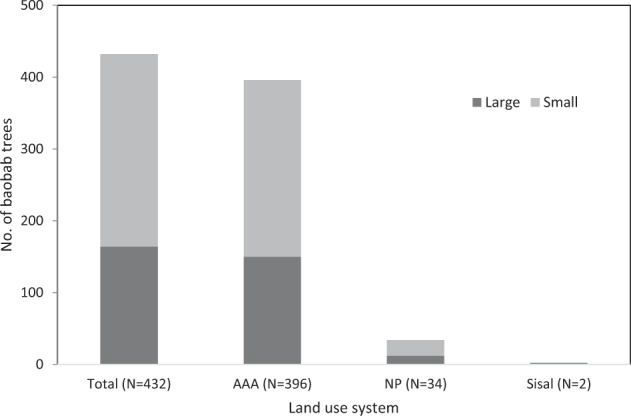
The number of large (DBH ≥ 1 m) and small (DBH < 1 m) baobabs (*n* = 432) in the total sample area and the different land use systems (AAA Anthropogenically Affected Area, NP National Park, Sisal Sisal Plantations), in Taita–Taveta County, Kenya

The entire surveyed area had a higher number of small baobabs (*n* = 267) than large baobabs (*n* = 165). Regarding the three land use systems, a higher number of small than large baobabs were documented in the NP (small 22; large 12) and the AAA (small 245; large 151), while the Sisal only had two large baobabs, but no small ones (Fig. 4). Small baobab trees were not as evenly distributed among the transects as large baobabs. While small baobabs were found in only 7 of the 49 surveyed transects (2 in NP, 0 in Sisal and 5 in AAA), large baobabs could be found in as many as 17 transects (5 in the NP, 1 in the Sisal and 11 in the AAA). All transects containing small baobabs apart from one also contained large baobabs.

With regard to small baobabs, two different types could be identified. While most small baobabs (79%) constituted normal seedlings or baobab juveniles, 21% were ‘stunted’ baobabs. These were characterized by a very broad base, wrinkled bark and strange, slightly sprawling thick branch-like outgrowths (Fig. 3S). In comparison, the normal baobab seedlings had thin shoot-like growth with many small twigs (Fig. 3S). All stunted baobabs were found in the high-density area (Taita cluster 7.5% of all small baobabs were stunted; Taveta cluster 46% were stunted).

The mean density for all baobabs documented in the study was 0.06 baobabs/ha, however, with marked differences among the three studied land use systems and within transects of one land use system. The highest mean baobab density per land use system was 0.11 baobabs/ha in the AAA and the lowest 0.002 baobabs/ha in the Sisal (Table 1). The greatest range of densities in transects within one land use system was found in the AAA with 0–1.193 baobabs/ha (Table 1).

**Table 1:** The mean densities of baobab trees/ha in the three surveyed land use systems in Taita–Taveta County, Kenya (means calculated using the transect densities)

			All baobabs			Large baobabs (DBH ≥ 1 m)			Small baobabs (DBH < 1 m)		
Land use system	Area sampled (ha)	Total No. of baobabs	Mean density	SD	Max. density	Mean density	SD	Max. density	Mean density	SD	Max. density
AAA	3600	396	0.110	0.272	1.193	0.042	0.096	0.387	0.068	0.195	0.927
NP	2850	34	0.012	0.044	0.193	0.004	0.012	0.053	0.008	0.032	0.140
Sisal	900	2	0.002	0.005	0.013	0.002	0.005	0.013	0.000	0.000	0.000

Data shown for all baobabs and large (DBH ≥ 1 m) and small (DBH < 1 m) baobabs separately. Per land use system, standard deviation (SD) and maximum density transect per land use type are also given. The land use systems are the Anthropogenically Affected Area (AAA), the national park (NP) and the sisal plantations (Sisal)

When dividing the sampled baobabs per stratum into large and small trees, the highest density of large trees was found in the AAA with a mean of 0.042 and a maximum of 0.39 baobabs/ha, while the lowest density was found in the Sisal (mean 0.002 baobabs/ha, maximum 0.01; Table 1). A similar pattern was observed for small baobabs (highest density in AAA, lowest in Sisal). However, in the AAA, small baobabs showed higher mean and maximum density than large baobabs (Table 1). As the baobabs in the sample area were found to grow clustered, calculating a density for the whole research area or per stratum would create an erroneous image of even distribution. The high standard deviation of densities within and between the different strata (Table 1) showed that the strata themselves only had a limited effect on the baobab distribution pattern, and that other factors may play a more important role in the distribution. Therefore, a new division into high-density and low-density areas was made to better describe the baobab distribution in the research area.

### High- and Low-Baobab Density Area

We were able to identify two baobab clusters showing high baobab density (i.e., ≥0.08 baobabs/ha), one being in Taita northwest of the town Voi (Taita cluster) and one in Taveta, just northeast and southeast of Taveta town (Taveta cluster) (Fig. 2). These baobab cluster areas were referred to as the high-density areas, while all space in between and around is considered low-density area, where only few trees were found.

While as many as 418 baobabs were found in the two high-density area clusters, only 14 baobabs were found in the whole low-density areas, which had an area twice as large as the high-density area (Table 2). Between the two high-density area clusters there were slight differences in densities, the Taveta cluster having a higher density (0.437 baobabs/ha ± 0.511), than the Taita cluster (0.347 baobabs/ha ± 0.244). The standard deviation in the Taveta cluster was, however, a lot higher than in the Taita cluster, therefore indicating that in the Taita cluster the baobabs were more evenly distributed than in the Taveta cluster.

**Table 2:** Comparison between high- (≥0.08 baobabs/ha) and low-density areas (<0.08 baobabs/ha) of baobabs and between the two high-density clusters in Taita and in Taveta

	Count of baobabs	No. of transects	Area sampled (ha)	Density (baobab/ha)	SD
Low-baobab density area	14	42	6300	0.002	0.004
High baobab density area	418	7	1050	0.398	0.391
Taita cluster	156	3	450	0.347	0.244
Taveta cluster	262	4	600	0.437	0.511

The density was calculated as the quotient of the baobab count per transect and the area sampled. Total baobab counts, number and area of transects, mean baobab densities and standard deviation (SD) are shown separately per category

### Size-Class Distribution (SCD)

**Fig. 5:**
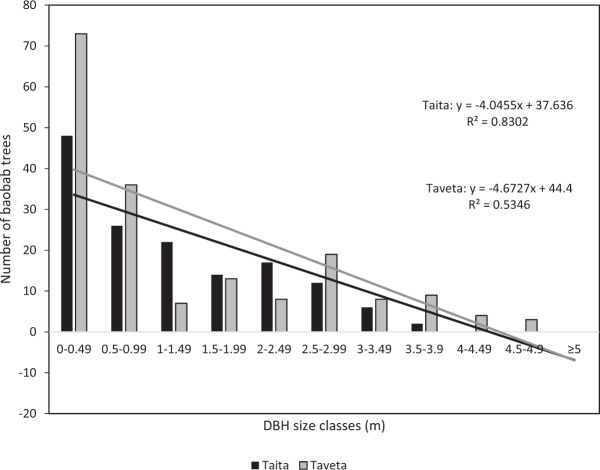
Size-class distribution curves based on the DBH (diameter at breast height in metres) of baobab trees in the two clusters in the high baobab density areas in Taita (150 documented baobabs where DBH could be measured) and Taveta (188 documented baobabs where DBH could be measured) in Kenya

The SCD of the two clusters in the high-density areas showed similar curves with a relatively high number of small baobabs and decreasing numbers of larger baobabs (Fig. 5). A comparison between SCDs of the high-density area and the low-density areas was not possible as there was not enough data from the low-density areas to fit a curve. The SCD curve of the high-density area cluster in Taveta was steeper than in Taita, indicating a higher number of smaller baobab trees. In addition, Taveta also had more trees in higher size classes (2.5 m and above), and more trees in the two lowest size classes (below 1 m) than Taveta, indicating that Taita had a more even distribution between the different size classes. The negative slope and higher presence of smaller rather than larger baobabs in both SCDs indicated a regenerative baobab population in both clusters (Fig. 5). The two SCDs of Taita and Taveta were not significantly different from each other (*p* = 0.13).

### Damage and Disease of Baobabs

Out of the 432 trees documented in the 49 transects, 75 trees (17%) showed signs of debarking by humans, elephants, insects and unknown entities (Fig. 3S). Most of the debarking was seen in Taveta (62 damaged trees), and the least in Taita (13 damaged trees). The most common debarking entity was said to be an insect. It was described by the Maasai people in the research area as a rose beetle-like insect called ‘Noongala’ in the local language, whose marks covered 44 of the total damaged 75 trees. The damage caused by the ‘Noongala’ beetle was difficult to evaluate as no insect was seen personally causing the damage during the research, and could therefore only be evaluated by the FGD in Taveta where the highest frequency of affected stunted baobabs was found. The same debarking marks were also observed on small trees of other species in the surrounding area (mainly *Commiphora* species). It was not possible to see damage on the larger baobab trees as the ‘Noongala’ beetle reportedly attacks branches on the tops of trees, these being too far off the ground for observation. While the ‘Noongala’ beetle was mentioned during FGDs and signs found during the baobab inventory only in the high-density area, respondents in the low-density areas reported a serious pest problem regarding termites. Termites were described as a highly destructive force, which attack all trees in their vicinity.

The most severe debarking was caused by elephants. However, the frequency of elephant damage (13 affected trees of the damaged 75 trees) was low and concentrated only in the NP and the directly surrounding areas.

Not included in the above mentioned documented debarking damages was the hollowing out of the baobab stems by humans for storage facilities, which was mentioned during the FGDs and during the household survey. However, this activity is no longer practised and no baobab with such a stem damage was found during the survey. On the other hand, stakes were often found hammered into the tree’s trunk for makeshift ladders, which, according to the respondents, are used for easy fruit harvesting and as escape routes against attacking wildlife.

### Occurrence and Perception of Baobabs on Farms and in the Surrounding Habitats

In the high-density areas, 16 of the interviewed 22 farmers (i.e., 73%) reported to have baobab trees on their farm. Each farm had a mean of 2 ± 1 baobabs according to the respondents. The baobabs were most commonly located in the farm boundary or the homestead area and were almost unanimously owned by the household head. No baobab tree had actively been planted by the surveyed households, although unintentional ‘sowing’ by fruit consumption and disposal of seeds was not excluded. Respondents mentioned that there is no need to plant baobabs as they grow wild in large numbers. All respondents knew how young baobab seedlings look like, and 11 out of 22 reported to have young baobabs growing on their farm. Most of the respondents (58%) would accept young baobabs growing on their farmland, while 42% of the respondents stated they would remove such seedlings. No respondent had ever cut down a baobab tree, and all referred to a law or taboo as a reason, or to the fact that cutting down a baobab would bring no benefits (such as firewood) and is labour and cost intensive. The baobab was most often perceived by the respondents as having a negative effect on crops (mainly concerning maize (*Zea mays*)) due to shading, nutrient uptake and high water use.

In the low-density areas, out of the 24 visited farms only 25% reported to have a baobab tree on their farm, which was mostly located in the farm boundary. Similarly to the high-density area, no respondent had ever planted a baobab tree. Occurrence of young baobab seedlings was reported to be very rare in the low-density areas. Out of the 24 respondents, 50% would protect such a seedling on their farm, while 50% would remove it. The perception of baobabs on farms was neutral, as there were few in the region. Trees in general were seen as positive by the respondents, however, not welcome near cropland due to shading and a believed water and nutrient leaching towards the tree roots. Most respondents (14 out of 22) claimed that they use the baobab trees that were not growing on their farms.

### Baobab Phenology and Use

**Fig. 6:**
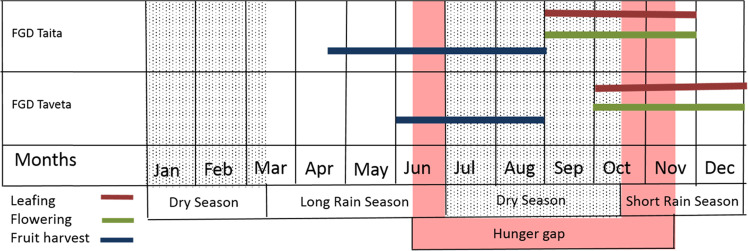
Seasonal calendar of baobab phenology according to results of focus group discussions in Taita (*n* = 8) and Taveta (*n* = 4), Kenya. The coloured bars indicate the different phenological stages of the baobab in the course of the year and the fruit harvest seasons. The pink area shows the time of the ‘hunger gap’ (time of high food insecurity levels) as mentioned by the respondents of the individual household survey. Source of the rain season data: NDMA (2014)

According to the FGD in the two clusters of high-density areas, baobab flowers and leaves appeared at the same time, in September in Taita and in October in Taveta, which is around the end of the dry season and the beginning of the short rainy season (due to this timing the respondents named the baobab the ‘rain making tree’ as the leaves mark the arrival of rains) (Fig. 6). Flowers as well as leaves are then said to be present on the trees for 3 months during the entire rainy season. The participants of the FDGs reported that about 7 months after the start of flowering, the first fruits are ready for harvest. In Taita, mature fruits are available for harvest from mid-April to end of August, while in Taveta from beginning of June to end of August, according to the respondents (Fig. 6).

For the local communities, baobab does play an important role as an emergency coping mechanism in the area. The season when baobab fruits are consumed coincides with the hunger gap mentioned by the respondents of the household survey. The main baobab consumption period, which is similar to its harvest period, spans from May and goes up to September/October according to the respondents. The ‘hunger gap’ or time when the interviewed household has less food available, spans from May/June to November/December (Fig. 6).

In total 27 different uses of baobab were recorded during the 46 individual household surveys and the 12 FGDs. The respondents mentioned the use of six different tree parts including the baobab fruit pulp, leaves, bark, roots, shell of the fruit and wood. In both the individual interviews and the FGDs more uses were mentioned in the high-density areas than in the low-density areas. The knowledge of uses, especially when considering the low-density areas, seemed to be shared more often amongst women than men. However, out of the 27 different uses mentioned, food use was the only use of baobab still actively practiced by the respondents in the research area.

The main reason given for consuming baobab fruit pulp during the household interviews differed. For some respondents it was its sweet taste (54% of the 46 respondents mentioning this) and the second most frequent reason was health (18%). Health benefits from baobab were described with comments such as: ‘It makes me feel like I have more blood’. Some respondents (13%) mentioned culture and tradition as reason for eating (‘because the grandparents ate it’) and 10% just the availability of trees in the vicinity. The mode of consumption differed between the surveyed areas. In high-density areas, most respondents reported to eat the raw pulp directly from the fruit, while in low-density areas, most respondents stated that they eat mabuyu sweets (i.e., pulp-covered baobab seeds cooked with sugar and food colour). During the household survey as well as the FGDs in the high-density areas, baobab was mentioned as being important as food during the dry season.

Respondents claimed that baobab is a ‘poor man’s food’ and ‘food for children’. The main consumers of baobab were finally named as children, herders and pregnant women.

### Perceptions of Changes in Baobab Populations and Factors causing these Changes

In the high-density areas, participants of the FGDs stated that the baobab population is either increasing due to large numbers of small baobabs growing or at least staying the same due to the extremely slow growth of such young baobabs, while some large baobabs may die at the same time. In these areas of high baobab densities, both small and large baobabs were observed (see Table 1), and the participants of all FGDs could describe young baobabs and where they could be found. Participants also highlighted that young baobabs take a long time to grow and that all changes in baobab populations tend to occur slowly mainly because of the dormancy period of the seeds, the recent lack of rainfall and the already mentioned slow growth rate. The participants agreed that seedlings generally tend to germinate in large numbers after every rainy season, but since they need 2–3 months of constant and reliable rainfall to establish and survive the next dry season, participants mentioned that baobab seedlings usually only survive if they had germinated after the more reliable short rainy season (October–December).

In the high-density areas, participants of the FGDs described several factors that could be responsible for changes in baobab populations. Consumption of fruits was given as an increasing factor, as it promotes seed dispersal. Wildlife was mentioned as both increasing factor (seed dispersal) and decreasing factor (browsing of young baobabs). Factors given by the participants that could negatively affect the baobab population in the high-density areas included construction and infrastructure—particularly related to the new Voi–Taveta highway that was being built in the area during the survey. Farming, agricultural expansion and population increase were also mentioned as decreasing factors as were climate change (defined as changing and unreliable rainfall patterns) and drought.

During the six FGDs performed in the low-density areas, participants stated that the baobab populations in their area were either staying the same due to a lack of seedlings or decreasing as a result of the missing seedlings and the death of old trees. It was also mentioned that baobabs in the low-density areas often did not bear fruit and that the few fruits were of low quality (small, hard fruits with bitter tasting pulp). The FGD participants in the low-density areas agreed that only few seeds may germinate and that the seedlings do not survive the dry season.

In the low-density areas, participants of the FGDs could not give any potential factor causing increasing baobab populations, but they described several factors that could be responsible for a decrease in baobab populations. Bush fires and browsing of small baobabs by livestock and wildlife were mentioned as the main decreasing factors, as well as human impact, mainly referring to the stakes hammered into the baobab trunks for easy climbing that may cause rotting and death of large baobabs according to the FGD participants. Similarly to the high-density areas, FGD participants in the low-density areas listed religion and beliefs in ‘djinnis’ as well as the law on protecting large trees as factors for decreasing baobab populations. The participants mentioned that farmers may remove any young baobab they find on their land before it can grow into a larger tree.

FGD participants of both high-density areas and low-density areas described religion/traditional beliefs and law as both increasing and decreasing factors in the baobab population. The traditional beliefs state that there is a djinni or other form of spirit living inside the baobab trees. The presence of the djinni on the one hand preserves the baobab since—according to the participants—terrible things are said to happen if the baobab is cut down. On the other hand, the presence of the djinni is not wanted, therefore, increasing the incentive to remove young baobabs before they reach a certain size. The law was described by the FGD participants as protecting trees after they have reached a certain height, after which removal is forbidden, thus enforcing the removal of baobab seedlings from farmland.

## Discussion

### Baobab Distribution in the Three Surveyed Land Use Systems

The fact that few baobabs were found in the land use type ‘Sisal’ holds true to the initial hypothesis that due to mechanization and intensive mono-cropping in the ‘Sisal’ (MEMR 2009), baobabs would not be found in high numbers. However, the hypothesis that there are much higher numbers of baobab trees in the NP due to low anthropogenic disturbance of the potential baobab populations was not confirmed. There are three main reasons why the NP could have less baobab trees than expected: (1) environmental factors, mainly water availability, (2) wildlife pressure, and (3) bush fires (Leuthold 1996; Cuni-Sanchez et al. 2010). Environmental factors may have played a major role as the area is extremely dry (average of 300–400 mm annual rainfall with very low levels of reliability; Jaetzold and Schmidt 1983) and has few sources of water (Smith and Kasiki 2000). Wildlife, however, also plays an important role as Leuthold (1996) found that the baobab population had basically been eliminated by elephants during the drought of the 1970s in the Tsavo NP. Leuthold’s (1996) study confirmed previous findings by Myers (1973), who concluded that it is highly likely that the baobab population would not be able to recuperate from the sustained damage. There are many grazers and browsers (including elephants) inside the NP who would also destroy small seedlings through trampling and browsing (Venter and Witkowski 2013a, b). Similarly, the livestock, which was brought into the NP by herders, caused large-scale overgrazing and degradation of the park’s natural resources (Waweru and Oleleboo 2013) including small baobabs. Another reason for the lack of baobab trees in the NP could also be due to fire, which can also greatly influence the baobab population (Cuni-Sanchez 2010). Fires would mainly affect small baobabs as large baobabs are usually able to survive due to the high water content in the stems (Gebauer et al. 2002). Respondents of our study described seasonal burning of pastures, occasionally expanding into the NP. This could cause the immediate destruction of small baobabs and—if occurring frequently—the slow destruction of large baobabs. However, similar to the study of Mpofu et al. (2012), who also described fire as a possible factor of baobab population reduction in Zimbabwe, no signs of fire were seen in the surveyed area, and therefore, the reasoning presented here is speculative.

In contrast to the NP, the AAA had more baobabs than expected in the present study, despite its high levels of anthropogenic disturbance. The AAA also showed a high variance in its baobab densities as it contains both high-density areas and low-density areas. More baobabs in the AAA could be attributed to a higher number of seed dispersers, such as humans, who consume baobab. Duvall (2007) stated that human habitation is important for baobab distribution, as he found a connection between baobab stands and human settlements. The people interviewed in the study region also confirmed that humans were one of the largest seed dispersers through their consumption of the baobab fruits. Other authors have also noted the importance of humans to baobab populations in countries such as Sudan, Malawi, South Africa and Burkina Faso (Cuni-Sanchez 2011a; Schumann et al. 2012; Venter and Witkowski 2013a, b; Wiehle et al. 2014). However, due to the high standard deviation and the clustered appearance of the baobab distribution in the studied land use type AAA, additional factors other than land use are assumed to play an important role.

### High- and Low-Density Areas of Baobab Occurrence

The baobabs in the surveyed area formed two distinct clusters at either end of the sample area. The tendency of baobabs to form clusters, or to be present in high and low densities within a confined area, was also reported from other countries such as Malawi and Mali (Dhillion and Gustad 2004; Cuni-Sanchez 2011a). Similarly, the high variability of baobab densities in the surveyed transects within the high-density areas (0.398 ± 0.391 baobabs/ha) is comparable to the ranges described by other authors from Mali, Sudan, Burkina Faso and Niger (Dhillion and Gustad 2004; Jensen et al. 2011; Gebauer and Luedeling 2013; Venter and Witkowski 2013a, b).

In the low-density areas of the Kenyan study site, environmental factors are assumed to be responsible for the low-baobab density (average 0.002 baobabs/ha). Respondents mentioned that the baobabs in the area continuously produced few to no fruits, therefore also severely reducing the number of seeds for dispersal. In addition, respondents mentioned that the fruits produced in the low-density areas had a bad taste, and were bitter and hard, therefore reducing their consumption. Due to the low consumption and fruit production, the seeds were never dispersed very far from the tree where they originated, leading to a very local seed dispersal, explaining the cluster formation. Seed viability in the soil seedbank would also decrease with time and predation (Venter 2012). Why fruit production is compromised in this part of the study area is unknown. Reasons mentioned from other countries include a lack of pollinators, too large distances between baobabs to allow pollinators to visit separate trees, or ‘wrong’ pollinators leading to self-pollination (Venter 2012). Self-pollination is not an advantageous occurrence since baobab is self-incompatible (Baum 1995). Recent studies also assume that genetic deviations or a kind of functional sexual dimorphism could cause poor fruit production in baobabs (Venter and Witkowski 2019). The low-density areas correspond to the drier (MEMR 2009; Smith and Kasiki 2000) and higher altitude areas in the study region (Taita and Sagalla hills) (Smith and Kasiki 2000), thus being rather a marginal habitat for baobabs. The current sparse distribution of baobabs and the lack of small baobabs found in the low-density areas of the study area could indicate that these were remnants of a relict population (Sidibe and Williams 2002a, b) from a more humid past. Further reasons for the low-baobab density in the low-density areas could be wildlife damage around the NP or pressure from agricultural expansion, livestock and slash-and-burn agriculture, which have been described as decreasing to the baobab population in South Africa and Malawi (Venter 2012; Cuni-Sanchez 2010; Wickens and Lowe 2008).

Heterogeneity of landscapes has been observed as conserving many different plant species and species richness in general (Bennett et al. 2006) of which baobab may also benefit. The landscape of the Taita cluster can be described as a mosaic landscape, mixing cropland and fallow areas, and had an observable higher human population density. This could aid in protecting and conserving baobab through lesser burning incidence (not common in mosaic landscapes), as well as providing a higher seed dispersal through more people consuming baobab fruits.

### Rejuvenation of Baobab Populations and Biotic Threats to Trees

In the surveyed area, small baobabs were found in a more constrained area than large baobabs. Due to climate change there will be changes to the ecological niche of baobab. Potentially the niche could shift, e.g., towards regions with more reliable rainfall and/or shrink, if no shift of niche is possible (Birhane et al. 2020). Both scenarios may pose a threat to the baobab with its frequently observed episodic recruitment (Schumann et al. 2010). In the low-density areas and at the edges of the clusters, small baobabs were almost completely missing.

Similar to the study of Gebauer and Luedeling (2013) in Sudan, the present study found an inverse j-curve of baobab size classes in the high-density areas and therefore indicated continuous recruitment and thus healthy baobab populations at least in the two clusters. Many baobab population studies in SSA, however, found a lack of rejuvenation (Venter and Witkowski 2013a, b; Dhillion and Gustad 2004; Cuni-Sanchez et al. 2010). In Mali, for example, Dhillion and Gustad (2004) found a bell-shaped distribution curve, showing a very low number of young baobabs. Although the lack of young baobabs was explained by faster growth of young trees, our findings contradict this as we found many small trees. Venter and Witkowski (2013a, b) explained the identified positively skewed and bell-shaped SCD curves by episodic recruitment, apparently common in long-lived species.

A high proportion of small baobabs in the study region was stunted, particularly in the Taveta cluster (86 out of the recorded 183 small baobabs). It is not clear if the stunted growth of small baobabs in Taveta has a negative effect on the population health of baobabs in this area. The reason for the stunting of baobabs is not clear. The local Maasai respondents attributed the stunting to damage caused by a rose beetle-like insect called ‘Noongala’. Possible candidates that could be harming the baobab trees are the longhorn beetle species *Analeptes trifasciata* Fabr., or *Paranaleptes reticulata* Thoms. (Fig. S4), both known for girdling trees, the latter being present in Kenya and known for causing damage on baobab (Jones 2015). Similar damage of baobab and cashew trees has also been observed in West Africa (Niassy et al. 2011; Sidibe and Williams 2002a, b). However, it is unsure whether these beetle species occur in Taita–Taveta and during the course of the present study, no beetles were observed damaging the bark of baobab trees.

The stunting of baobabs could also be attributed to the high numbers of livestock reared by the local Maasai people in the area. A similar damage of baobabs was described by Dhillion and Gustad (2004) and Venter (2012) in high livestock areas, caused by frequent browsing of leaves and young branches. A further explanation was given by FGD respondents in the Taveta area, who claimed that stunted baobabs were not normal seedlings but sprouts of older baobab tree roots. However, these statements could not be proven (e.g., by digging out stunted baobabs) during the study.

Activities such as leaf harvest, bark harvest and intensive fruit harvest can also result in negative effects on baobab populations (Schumann et al. 2012; Venter and Witkowski 2011; Gebauer and Luedeling 2013). In Taita–Taveta, however, leaves were only mentioned as being used for medicine and fodder but not for food, putting the sample area in contrast with many West African countries where baobab leaves are part of the daily diets (Buchmann et al. 2010). Similarly, the removal of bark was described only by few respondents of the FGDs and mostly old marks of bark harvest were found, probably because nowadays cheap plastic ropes and baskets or bags are easily available at the local markets. The proportion of debarked baobabs in the sample area can be rated as low (only about 17% of trees affected) compared with other countries such as Malawi where >50% of all baobabs were debarked (Cuni-Sanchez 2011b).

Consistent fruit harvest in large amounts according to the respondents was not common in many areas of the surveyed area, and therefore may not constitute a large problem for rejuvenation of baobab. Often, single trees remain unharvested, while for others, due to simple harvesting practices (throwing stones against the fruits or climbing the tree), some fruits remain on the tree (Jäckering et al. 2019). This could, however, also be due to varying tastes of fruits or other reasons such as the height of the tree making harvesting more difficult. In general, baobab trees did not seem to be highly exploited or overused in Taita–Taveta as only the fruits were used for food or processed to mabuyu sweets.

### Importance of Baobab for Food Security

Baobab was an important food source for the most vulnerable members of studied rural communities, but was at the same time perceived as ‘poor man’s food’. Due to their naturally dry fruit pulp, undamaged baobab fruits can be stored for several months and can therefore be used whenever needed for home consumption or sale in emergency situations. The seasonal phenological patterns documented in the FGDs in Taita–Taveta differ slightly from those produced by Maundu (1999) in Kibwezi area and the coast of Kenya. However, such slight differences are normal as climatic conditions, particularly the onset of the rainy season also differed between the mentioned regions of Kenya. In general, the concurrent occurrence of baobab flowers and leaves at the usual beginning of the rainy season was also recorded in South Africa (Venter and Witkowski 2019). The time mentioned as the ‘hunger gap’ by the respondents of the present study covered most of the harvest and consumption periods of baobab (Fig. 5). Through this baobab had a great potential to be used as emergency food and help to overcome food-insecure periods, particularly for children. In addition, it was a source of income in the region (Jäckering et al. 2019) as described similarly from other areas in SSA (Adam et al. 2013; Gebauer and Luedeling 2013; Kehlenbeck et al. 2013; Venter and Witkowski 2013a, b).

The leaves also have the potential to be a highly nutritious and healthy food for humans, due to their high micronutrient content (Chadare et al. 2009). However, use of fresh or dried leaves as a vegetable was unknown in the research area, where the most important and most frequent food use mentioned referred to the fruit pulp and the most common consumers were children, pregnant women and herders.

## Conclusion

Many areas of SSA have reported ageing baobab populations with a lack of rejuvenation. These findings coupled with the possibilities of a growing export market for baobab fruit pulp have generated concern over the long-term stability of baobab populations in SSA. In Taita–Taveta County, we documented rejuvenating baobab populations in two areas with high baobab densities, but also large areas in between with low densities and no regeneration. The patterns in baobab distribution were attributed to environmental factors, wildlife interactions and human impact. Almost all small baobabs in one of the high-density areas were stunted, seemingly due to high livestock pressure and damage by a tree girdling longhorn beetle. This could have consequences for the long-term stability of the population. In the research area, baobab serves as an important emergency food during the ‘hunger gap’ period. However, baobab food products were mainly perceived as poor man’s food and as food for children. Use levels of baobab were therefore rather low and local communities mainly used raw or slightly processed fruit pulp while many of the traditional further uses were forgotten.

The generally healthy status of the baobab populations with high numbers of small baobabs and good recruitment rates in parts of the research area together with the detected rather low level of use of baobab products lead to the conclusion that this resource could be used in a more intensive way. Such a sustainable intensification of baobab resource use could also be a measure to conserve plant genetic resources. In low-baobab density areas, planting and actively managing baobabs should be supported if communities want to benefit from the nutrient-rich products and to participate in the baobab business. In the areas with high densities, baobab has already shown a certain potential for improving local livelihoods in Taita–Taveta County. It is ripe during the food scarce time and could provide the local community with nutritious foods and additional income. However, awareness of communities should be raised on additional nutritious food products of baobab such as leaves. If economically viable and marketable in the larger region, forgotten traditional and innovative new processed food products such as baobab juice, soda, yoghurt or ice cream could also be promoted.

Intensification of baobab utilization in Taita–Taveta, has to be done with care. Baobab pulp is currently being used within the local community, particularly by children and may contribute substantially to their nutrition, particularly during the lean season. Therefore, it would be prudent to focus most attention on creating a local demand and encouraging the use of baobab on a local scale prior to entering larger or even export markets. It would also be advisable to integrate the traditional resource management techniques and traditional values into future programmes on increased production and marketing of baobab products. In the long run, only domestication and cultivation of improved baobab cultivars may be able to deliver the required qualities and quantities for both domestic and export baobab fruit pulp markets.

## Supplementary Information

Supplementary Material
