# Correction to: The Baobab (*Adansonia digitata* L.) in Southern Kenya–A Study on Status, Distribution, Use and Importance in Taita–Taveta County

**DOI:** 10.1007/s00267-021-01490-x

**Published:** 2021-06-09

**Authors:** Sahrah Fischer, Lisa Jäckering, Katja Kehlenbeck

**Affiliations:** 1 Institute of Agricultural Sciences in the Tropics (Hans-Ruthenberg-Institute), University of Hohenheim, Garbenstr. 13, 70599 Stuttgart, Germany; 2 International Fund for Agricultural Development (IFAD), Via Paolo di Dono, 44 00142 Rome, Italy; 3 World Agroforestry Centre (ICRAF), United Nations Avenue, Gigiri, PO Box 30677, Nairobi, 00100 Kenya; 4 Faculty of Life Sciences, Rhine-Waal University of Applied Sciences, Marie-Curie-Straße 1, 47533 Kleve, Germany

Correction to: *Environmental Management* (2020) 66:305–318

10.1007/s00267-020-01311-7

The article “The Baobab (*Adansonia digitata* L.) in Southern Kenya–A Study on Status, Distribution, Use and Importance in Taita–Taveta County” written by Sahrah Fischer, Lisa Jäckering and Katja Kehlenbeck was originally published Online First without Open Access. After publication in volume 66, issue 3, page 305–318 the author decided to opt for Open Choice and to make the article an Open Access publication. Therefore, the copyright of the article has been changed to © The Author(s) 2021 and the article is forthwith distributed under the terms of the Creative Commons Attribution 4.0 International License, which permits use, sharing, adaptation, distribution and reproduction in any medium or format, as long as you give appropriate credit to the original author(s) and the source, provide a link to the Creative Commons licence, and indicate if changes were made. The images or other third party material in this article are included in the article’s Creative Commons licence, unless indicated otherwise in a credit line to the material. If material is not included in the article’s Creative Commons licence and your intended use is not permitted by statutory regulation or exceeds the permitted use, you will need to obtain permission directly from the copyright holder. To view a copy of this licence, visit http://creativecommons.org/licenses/by/4.0. Open access funding enabled and organized by Projekt DEAL.

The original article has been corrected.

