# Supplementary material for: The Baobab (Adansonia digitata L.) in Southern Kenya–A Study on Status, Distribution, Use and Importance in Taita–Taveta County
Source: Environ Manage. 2020 Jun 12;66(3):305–18. doi: 10.1007/s00267-020-01311-7 (PMC8172389; doi:10.1007/s00267-020-01311-7)
Supplement: Supplementary file 1 — Supplementary Material [file 267_2020_1311_MOESM1_ESM.docx]

SUPPLEMENTARY MATERIAL


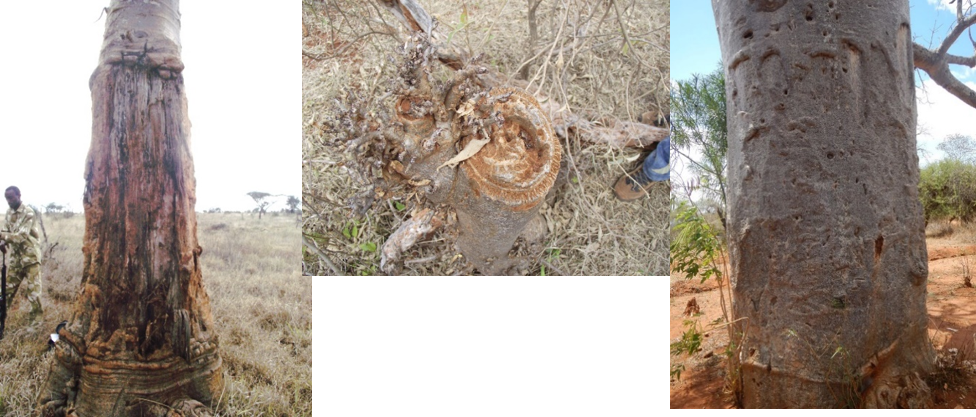


(a)

(b)

(c)

Figure 1S. Different types of debarking damages on baobab in Taita-Taveta County, Kenya. (a) Elephant marks (damage level: 3) (photo by Sahrah Fischer); (b) Insect damage (damage level: 3-4) (photo by James Hunter); and (c) Human damage (damage level: 1) (photo by Lisa Jaeckering).


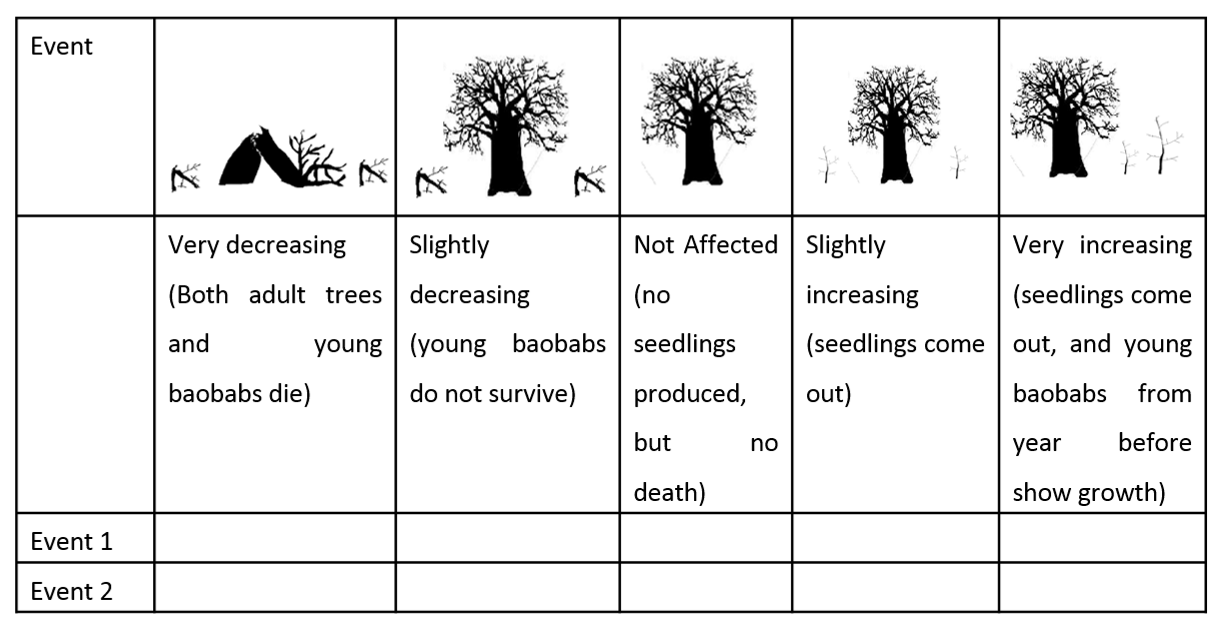


Figure 2S Set up of the occurrence game used in the FGDs. Event 1 and Event 2 would represent events mentioned by the respondents (e.g. drought, construction work) affecting the baobab population.


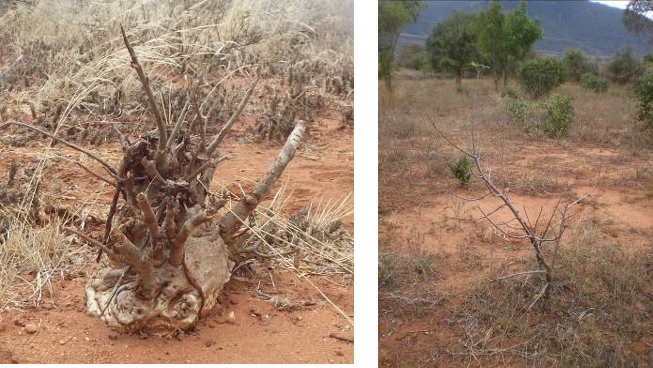

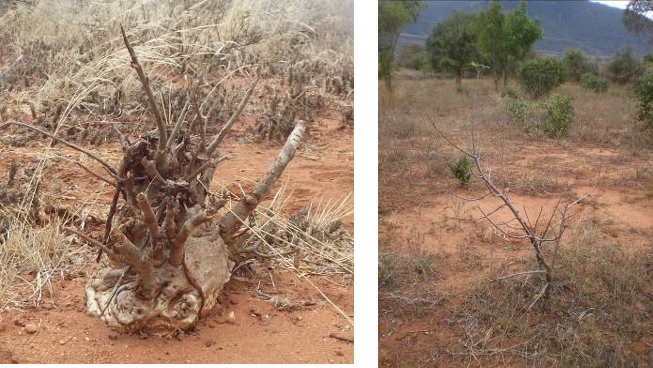


**(a)**

**(b)**

Figure 3S: The two different types of small baobabs found in the research area in Taita-Taveta County, Kenya. (a) Stunted baobab with a broad base, (b) normal baobab seedling.


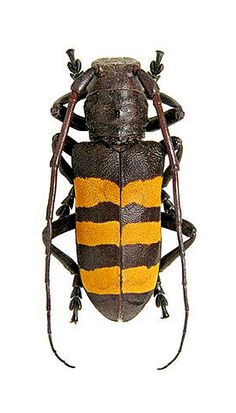

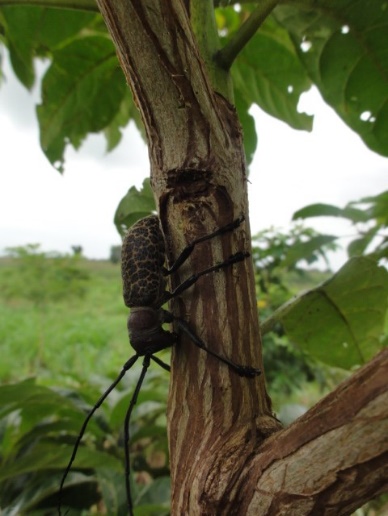


Figure 4S The images show possible candidate species of longhorn beetles responsible for the observed damage done to small stunted baobab trees in Taita-Taveta County, Kenya. The left image shows *Analeptes trifasciata* Fabr (source: <https://www.pinterest.de/jfnhr/insekt/>, Access: 25.10.19), the right image shows *Paranaleptes reticulata* Thoms (Source: <https://www.cfwt.sua.ac.tz/forestrybiology/index.php/84-latest-news/175-a-tree-girdling-beetle-paranaleptes-reticulata-thoms-in-forest-plantation-and-woodlots-of-tanzania>, Access: 25.10.19)


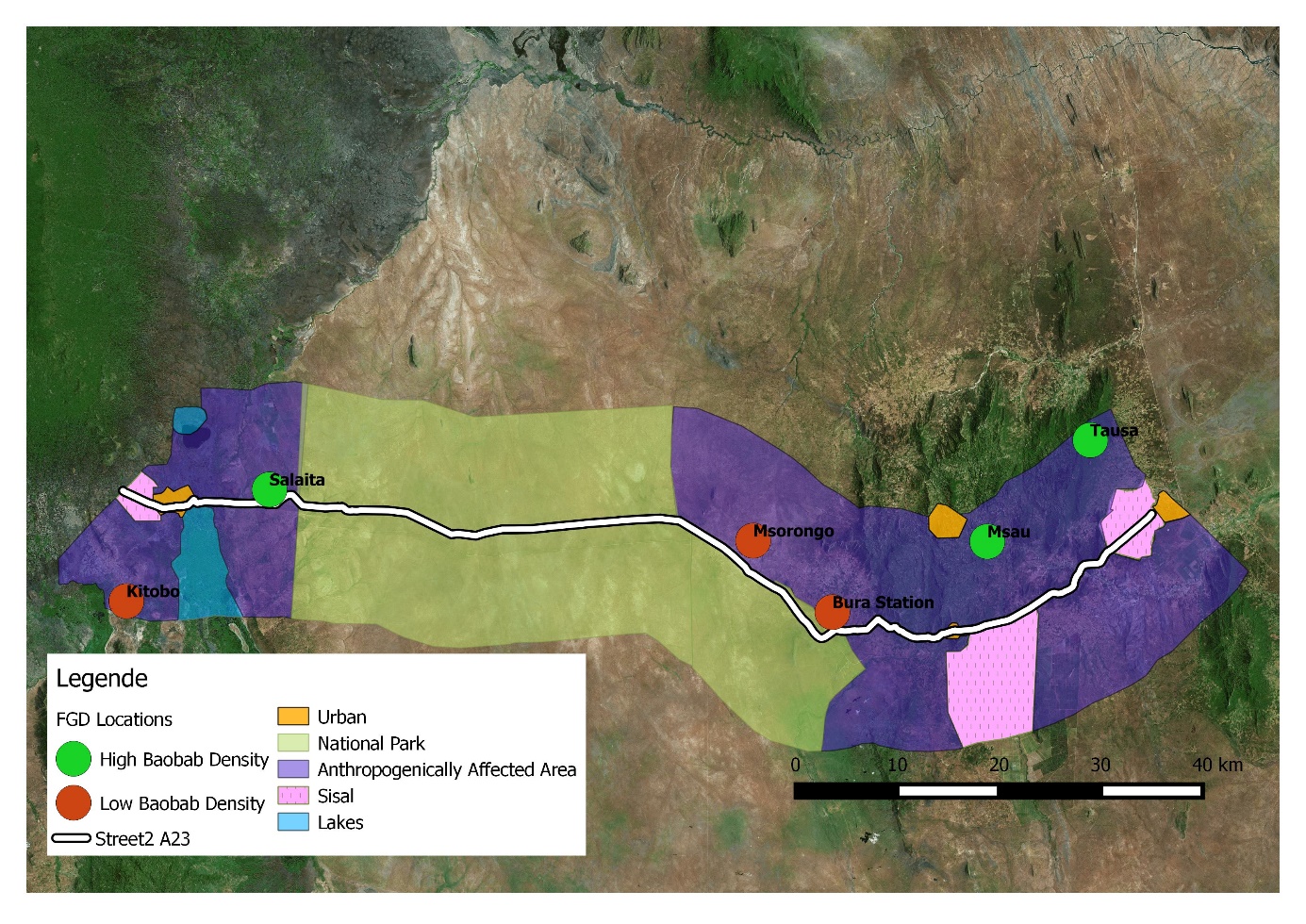


Figure SX The locations of the focus group discussions in Taita Taveta, Kenya, on baobab distribution, use and population changes in high (i.e. ≥ 0.08 baobabs/ha) and low baobab density areas (< 0.08 baobabs/ha). Colours show the three different land use systems sampled.

METHODS S1 – Baobab transect survey


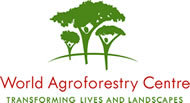


Baobab Transect Survey in Taita-Taveta, Kenya

Transect No. : ……………………………………..

Date of Walk: ………………………………………

Start point along middle line (S°, E°): ………………………………………………………………………………………………

End point along middle line (S°, E°): …………………………………………………………………………………………………

Four corners of the transect (S°, E°) (1) ……………………………………………………….. Altitude (m asl) ………………………………

(2) ……………………………………………………….. Altitude (m asl) ………………………………

(3) ………………………………………………………… Altitude (m asl) ………………………………

(4) ………………………………………………………… Altitude (m asl) ………………………………

Notes on the region:

Transect table (see footnotes for term, code and method explanations):

| Baobab code and variety | Tree no. 1 | Tree no. 2 | Tree no. 3 | Tree no. 4 |
| --- | --- | --- | --- | --- |
| Location S° |  |  |  |  |
| Location E° |  |  |  |  |
| Altitude  (and error) |  |  |  |  |
| Major landform |  |  |  |  |
| Topographic position |  |  |  |  |
| Land cover |  |  |  |  |
| Land use |  |  |  |  |
| Soil |  |  |  |  |
| Image code |  |  |  |  |
| Trunk CBH (m) |  |  |  |  |
| Tree height (m) |  |  |  |  |
| Crown width (m) |  |  |  |  |
| Occurrence of debarking (old or new) |  |  |  |  |
| Occurrence of diseases or pests |  |  |  |  |
| Flower presence/  stage |  |  |  |  |
| Fruit presence/  stage |  |  |  |  |
| Leaf presence/ stage |  |  |  |  |
| Bark colour |  |  |  |  |
| Undergrowth (descriptive) |  |  |  |  |
| Land Ownership |  |  |  |  |
| Other notes |  |  |  |  |

Coding (*):

- Major Landform: Level, Sloping, Steep, Composite (Vagen et al. 2013)
- Topographic Position:
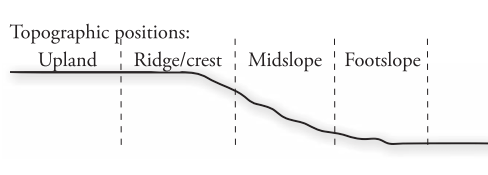
 taken from (Vagen et al. 2013)
- Land Cover: Forest, Woodland, Bushland, Thicket, Shrubland, Grassland, Wooded Grassland, Cropland (adapted from (Vagen et al. 2013) – definitions from most of the indicators taken from there.

| **No.** | **Type** | **Description** |
| --- | --- | --- |
| 1 | Forest | A continuous stand of trees, their crowns interlocking. |
| 2 | Woodland | An open stand of trees with a canopy cover of 40 % or more. The field layer is usually dominated by grasses. |
| 3a | Bushland | A mix of trees and shrubs with a canopy cover of 40% or more |
| 3b | Thicket | A closed stand of bushes and climbers usually between 2 and 7 m tall |
| 4 | Shrubland | An open or closed stand of shrubs up to 3 m tall 5 |
| 5 | Grassland | Land covered with grasses and other herbs, either without woody vegetation or the woody cover is less than 10 % |
| 6 | Wooded Grassland | Land covered with grasses and other herbs, with woody vegetation covering between 10 and 40 % of the ground |
| 7 | Cropland | Cultivated land (or being prepared for cultivation (if sampling in the dry season)) with annual or perrenial crops |

- Land use: Urban, Village land, Small-scale Agriculture, Fallow land, Rangeland , Boundary, Other
- Debarking: 0=no damage; 1=slight damage with few scars; 2=moderate damage with numerous scars; 3=severe damage with the tree scarred deeply; 4=dead tree (also add E=Elephant; H=Human); also add whether its recent or old (Mpofu et al. 2012)
- Topography: is there a slope, approximate degrees, sun or not and direction
- Presence of fruit, leaves: Y/N and if yes described using the draft of the descriptors for baobab (Kehlenbeck et al. 2015)
- Soil: following the AfSIS Technical Specification, 2010 pg. 8 fig. 7
- Land ownership: Private, Communal, Government, Don’t Know (Vagen et al. 2013)

Arzai, A.H. & Aliyu, B.S., 2010. THE RELATIONSHIP BETWEEN CANOPY WIDTH , HEIGHT AND TRUNK SIZE. *Bayero Journal of Pure and Applied Science*, 3(1), pp.260–263.

Baum, D.A., 1995. A Systematic Revision of Adansonia (Bombacaceae). *Annals of the Missouri Botanical Garden*, 82(3), pp.440–471.

Kehlenbeck K, Padulosi S, Alercia A. 2015. Descriptors for Baobab (*Adansonia digitata* L.). Bioversity International, Rome, Italy and World Agroforestry Centre, Nairobi, Kenya.

Mpofu, E. et al., 2012. Abundance , distribution and status of African baobab ( Adansonia digitata L .) in dry savanna woodlands in southern Gonarezhou National Park , southeast Zimbabwe. *Tropical Ecology*, 53(1), pp.119–124.

Powell, D.C., 2005. HOW TO MEASURE A BIG TREE. *Umatilla National Forest*, pp.1–9.

SCUC, 2006. *Baobab Adansonia digitata - Practical Manual No. 4 - Field Manual for Extension Workers and Farmers*, Southampton: University of Southampton.

Sidibe, M. & Williams, J.T., 2002a. *Baobab Adansonia digitata L.* A. Hughes, N. Haq, & R. W. Smith, eds., Southampton: International Centre for Undertilised Crops.

Sidibe, M. & Williams, J.T., 2002b. *Fruits for the Future 4 - Baobab Adansonia digitata L.*, Southampton, UK.: International Centre for Undertilised Crops.

Vagen, T.-G. et al., 2013. *the Land Degradation Surveillance Framework - LDSF Field Guide*, Nairobi: World Agroforestry Center(ICRAF) and International Centre for Tropical Agriculture (CIAT).

**Methods S2 – Household Survey Questionnaire**


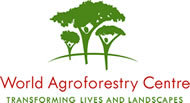


TAITA-TAVETA BAOBAB FRUIT PILOT STUDY

FARMER QUESTIONNAIRE

**Questionnaire No. Altitude (m asl) /GPS**

**Date of Interview: Transect and Baobab(s) Code**

**INTRODUCTION**

The World Agro-forestry Centre (ICRAF) is conducting a pilot study on Baobab Fruit trees in the Taveta area. Your participation is important since it will help in providing crucial data for future benefit of the baobab industry in Kenya. Please note that your response will be used for statistical analysis only and shall be treated confidentially.

Name of the Respondent: …………………………………………………………………………..

Respondent’s code: …………………………………………………………………………………….

Sex of the respondent: Male { } Female { }

Age of the respondent: …………………………………………………………………………………

Tel No. / Contact ……………………………………………………………………

Name of household head/ contact: ……………………………………………………………….

Enumerator :………………………………………………………………………………………………..

Supervisor: ……………………………………………………………………………………………………..

Location: ……………………………………………………………………………..

Division: …………………………………………….... District: ……………………………………………

Distance to the nearest market: ………………………………………………………….(km/time it takes)

Name of the market: ………………………………………………………………………………………..

1. **Socioeconomic information.**
2. **Household characteristics**

We would like to know a little more about who is living in your household.

| **HH Members** | **Name** | **Gender** | **Relationship to hh head** | **Marital Status** | **Age** | **Education Level** | **Tribe** | **Religion** | **Occupation** |
| --- | --- | --- | --- | --- | --- | --- | --- | --- | --- |
| 1 |  |  |  |  |  |  |  |  |  |
| 2 |  |  |  |  |  |  |  |  |  |
| 3 |  |  |  |  |  |  |  |  |  |
| 4 |  |  |  |  |  |  |  |  |  |
| 5 |  |  |  |  |  |  |  |  |  |
| 6 |  |  |  |  |  |  |  |  |  |
| 7 |  |  |  |  |  |  |  |  |  |
| 8 |  |  |  |  |  |  |  |  |  |
| 9 |  |  |  |  |  |  |  |  |  |
| 10 |  |  |  |  |  |  |  |  |  |

**Codes: Role** (1 Household head, 2 wife, 3 husband, 4 daughter, 5 son, 6 Grandparents)

**Codes: Education** (1.Never, 2. Some-primary, 3. Primary finished 4. Secondary finished 5. Tertiary)

**Code: Religion** (1. Muslim, 2. Christian, 3. Others)

**Codes: Tribe** ( 1.Wadawida (Taita), 2. Wasaghala (Sagalla), 3. Wataveta (Taveta), 4. Massa 5.other)

**Codes: Marital Status** ( 1. Single 2. Married 3. Divorced 4. Separated 5. Widowed)

x. Does your household live here all year round? Yes { } No { }

xi. If no: Specify: ………………………….

Code: 1. Nomadic 2.Seasonal residence

1. **Land Use**

We would like to know a little bit more about your farm/land.

1. What size is your farm in acre? ………………………………………..
2. Do you cultivate the whole area? Yes { } No { }
3. If no: Give proportion of uncultivated land

**……………………………………………………………….**

1. What is the current ownership status of your farm? ……………………………..

{1} Own with title {2} Own pending title {3} Rented {4} Tribal land {4} Other ……………………………………..

1. How many plots do you cultivate?......................................number
2. What crops do you grow in your farm? Please name which crops you grew during the last cropping season. List up to 3 per plot

| **Plots** | **Size in acre** | **Irrigated** | **Crops** | **How much did you harvest last season** | **How many sacks did you sell?** | **Market price last season per unit** |
| --- | --- | --- | --- | --- | --- | --- |
| 1. |  |  |  |  |  |  |
| 2. |  |  |  |  |  |  |
| 3. |  |  |  |  |  |  |
| 4. |  |  |  |  |  |  |
| 5. |  |  |  |  |  |  |

Codes: 1. Maize 2. Beans 3.Cowpea 4. Cassava 5. Cotton 6. Others (Specify)

……………………………………………………………………………………..

1. Do you rear livestock in your farm? Yes { } No { }

| **Livestock Type** | **Number (per head)** | **Permanent or migratory** | **How many did you sell last season?** | **Market price last season** |
| --- | --- | --- | --- | --- |
|  |  |  |  |  |
|  |  |  |  |  |
|  |  |  |  |  |
|  |  |  |  |  |
|  |  |  |  |  |

**Code:** Type: {1} Cattle {2} Goats / Sheep {3} Donkeys / Camels {4} Chicken / other birds {5} Other

1. **Tree Overview**
2. Do you have any trees on your farm? Yes { } No { }
3. If yes , please start with the most important ones (Are there any wild trees? Are they important as well?) Please list

| **Tree** | **Local name** | **Use** |
| --- | --- | --- |
|  |  |  |
|  |  |  |
|  |  |  |
|  |  |  |
|  |  |  |

1. **Baobab Trees**
   1. **On-Farm**

*We would like to talk more about the baobab trees now*

1. Do you have any baobab trees on your farm? Yes { } No { }

If yes, how many ……………………………………

1. Where are they?............................................

Code: 1. Homestead area; 2. Fallow field; 3. Cash crop; 4. Wood lot; 5. Boundaries

1. Who owns the baobab tree(s) on this farm? ………………………………………….
2. How old is the Baobab(s) on your farm?.........................................
3. How did the Baobab get on your farm? Planted { } Wild { }
   1. If planted: Who planted the baobab?.............................................

(1) Father (2) Mother (3) Respondent (4) Grandparents (5) Other (Specify)

- 1. If planted how did you plant it? Seed{ } Grown in a pot first { }
  2. What planting material did you use?

1. Seedling (2) Wildling (3) Cutting (4) Truncheons (5) Seed

...............................................................................

- 1. Where did you get the material from (if a nursery, then name and address)?

.........................................................................................

1. Has anybody ever planted a Baobab on your farm that didn’t survive?

Yes { } No { }

If yes, why?...........................................................................

1. When did you last see a young baobab?
   - 1. Never (1) Days (2) Weeks (3) Months (4) Years

v. Do you have young baobab trees on your farm?

Yes { } No { }

1. Do you know what a young Baobab looks like?

Yes { } No { }

1. What would you do if you would find a young Baobab on your farm?.............
2. Nothing (1) Protect and manage (weeding, watering, fertilizer) (2) Protect it from livestock (3) Frequent checks (manage without protection) (4) Infrequent checks
3. Do you only use the baobab trees that are on your farm?

Yes { } No { }

Codes: 1. Father 2. Mother 3. Own 4. Grandparents 5. Employer 6. Other (Specify)

………………………………………………………………………………………..

1. Have you ever cut down a Baobab on your farm? Yes { } No { }

If yes, why (wood, space, bad for crops)?..........................................................................

- 1. **Off-Farm**

1. In your lifetime, did the number of Baobab trees change?

(1) Increase (2) Decrease (3) Same Why? ………………………………………………..

1. Do you know of any baobab trees around your farm? Yes { } No { }

If yes, where/how long does it take to get there?.............................................

1. If yes, do you, or a member of your household use them? Yes { } No { }

If yes, who? ................................................

(IF NOT ANSWERED ABOVE)

1. Do you know what a young Baobab tree looks like?

Yes { } No { }

1. When did you last see a young baobab?
2. Never (1) Days (2) Weeks (3) Months (4) Years
3. Have you ever seen a young Baobab that was not on your farm?

Yes { } No { }

1. What would you do if you would find a young Baobab?.............
   - 1. Nothing (1) Protect and manage (weeding, watering, fertilizer) (2) Protect it from livestock (3) Frequent checks (manage without protection) (4) Infrequent checks
2. Have you ever cut down a Baobab that was not on a farm? Yes { } No { }

If yes, when and why (wood, space)?..........................................................................

1. Would you consider planting a Baobab? Yes { } No { }

If yes, why haven’t you planted one yet? List reasons

i)...................................................................

ii)..................................................................

iii)...................................................................

**General questions**

1. When do the following events occur in relation to baobab. Beginning with the nearest tree to the homestead:

GPS coordinate of closest tree:

| **Season** |  |  | **LRS** | **LRS** | **LRS** | **LRS** |  |  |  | **SRS** | **SRS** | **SRS** |
| --- | --- | --- | --- | --- | --- | --- | --- | --- | --- | --- | --- | --- |
| **Phenological Stage** | **Jan** | **Feb** | **Mar** | **Apr** | **May** | **Jun** | **Jul** | **Aug** | **Sept** | **Oct** | **Nov** | **Dec** |
| Leafing |  |  |  |  |  |  |  |  |  |  |  |  |
| Flowering |  |  |  |  |  |  |  |  |  |  |  |  |
| Fruiting |  |  |  |  |  |  |  |  |  |  |  |  |
| Harvest (Main Season)  - Fruit   - Leaves - Bark |  |  |  |  |  |  |  |  |  |  |  |  |
| Germination (Natural) |  |  |  |  |  |  |  |  |  |  |  |  |

LRS= Long Rain Season SRS= Short Rain Season

1. Do the different stages always happen at the same time, or is it different every year?

Same { } Different { }

1. Is the time of occurrence of each stage the same for all other trees around or does it differ?

Same { } Different { }

If different, and the respondent knows the phonological stages of the other trees record the stages in a new table.

1. **Utilization of Baobab**
2. What parts of the baobab do you use?

*Did you use any of the leaves. If yes: How often…*

| **No.** | **Baobab Part** | **Yes**  **No** | **Usage (e.g. fuel wood, shelter, food etc)** | **Who uses the part of the tree?** | **How often did you use this part in the past year?** | **When did you last use it?** |
| --- | --- | --- | --- | --- | --- | --- |
| 1. | Leaves |  |  |  |  |  |
| 2. | Flowers |  |  |  |  |  |
| 3 | Fruit |  |  |  |  |  |
| a | Shell (from fruit) |  |  |  |  |  |
| b | Fibers in the Fruit |  |  |  |  |  |
| c | Pulp |  |  |  |  |  |
| d | Seeds |  |  |  |  |  |
| 7. | Roots |  |  |  |  |  |
| 8. | Trunk |  |  |  |  |  |
| 9. | Bark |  |  |  |  |  |
| 9. | Others (Specify) [Include services of whole tree and beliefs associated with tree e.g. shade, mulch, soil improvement, border mark, bringing good luck etc.] |  |  |  |  |  |
| 10. |  |  |  |  |  |  |
| 11. |  |  |  |  |  |  |
| 12. |  |  |  |  |  |  |
| 13. |  |  |  |  |  |  |

Codes for Uses: 1. Food 2. Medicine 3. Browse 5. Fencing and Construction 6. Carving &

Crafts 7. Religion 8. Landmark 9. Other (Specify) ………………………………………

………………………………………………………………………….

1. How do you utilize your total baobab produce / products?

| **No.** | **Baobab Product** | **Who Does The Harvest?** | **Harvest season** | **Frequency of harvest during season** | **Harvest amount per harvest (Kgs / Sacks / Other [Specify] )** | **How much time do you spend per single harvest?** | **Out of total harvest: For home consumption (%)** | **Given Away as Gifts / Tithes (%)** | **Sold (%)** | **Other (%)**  **[Specify]** |
| --- | --- | --- | --- | --- | --- | --- | --- | --- | --- | --- |
| 1. |  |  |  |  |  |  |  |  |  |  |
| 2. |  |  |  |  |  |  |  |  |  |  |
| 3. |  |  |  |  |  |  |  |  |  |  |
| 4. |  |  |  |  |  |  |  |  |  |  |
| 5. |  |  |  |  |  |  |  |  |  |  |
| 6. |  |  |  |  |  |  |  |  |  |  |
| 7. |  |  |  |  |  |  |  |  |  |  |

Codes: 1. Father 2. Mother 3. Daughters 4. Sons 5. Grandparents 6. Employee 6. Other (Specify) ………………………………………………………………………………………..

1. **Specific questions for baobab fruits:**
2. What is the average harvest per single harvest?
3. Does the harvest differ between years?

Yes { } No { }

1. If yes: How and why?
2. Is there anything left on the tree?

Yes { } No {

1. If yes: How much is left (proportions) and please give reasons?
2. **Fruit Tree Planting & Management**
3. Are there any different types of baobab?

Yes { } No { } Don’t Know { }

1. If yes, please describe the different types: What are their characteristics?

| **No.** | **Variety (as known locally)** | **Distinguishing Characteristics** |
| --- | --- | --- |
|  |  |  |
|  | General |  |
|  |  |  |
|  |  |  |
|  |  |  |
|  |  |  |

1. What management practices do you provide the baobab fruit trees on your farm?

(e.g. Pruning, Weeding, Fertilizing, Manure application, De-fruiting, Pest & Disease Control etc)

| **Management Practice** | **Who does it?** | **How often?** | **How much time do you spend on doing it?** |
| --- | --- | --- | --- |
|  |  |  |  |
|  |  |  |  |
|  |  |  |  |
|  |  |  |  |
|  |  |  |  |
|  |  |  |  |

1. What management practices do you provide the baobab fruit trees that are not on your farm? (e.g. Pruning, Weeding, Fertilizing, Manure application, De-fruiting, Pest & Disease Control etc)

| **Management Practice** | **Who does it?** | **How often?** | **How much time do you spend on doing it?** |
| --- | --- | --- | --- |
|  |  |  |  |
|  |  |  |  |
|  |  |  |  |
|  |  |  |  |
|  |  |  |  |
|  |  |  |  |

1. What problems do you encounter in growing baobab trees and what are the possible remedies? (Separate table for wild and planted trees, first discuss the planted trees (if there are any) then the wild trees)

| **Problem** | **When** | **Where** | **Level of severity** | **Possible Solution** | **Ever tried?** |
| --- | --- | --- | --- | --- | --- |
|  |  |  |  |  |  |
|  |  |  |  |  |  |
|  |  |  |  |  |  |
|  |  |  |  |  |  |
|  |  |  |  |  |  |
|  |  |  |  |  |  |

1. **Processing Table**
2. Do you process your baobab products?
   - 1. Yes { } No { }
3. If no: Can you give reasons why you do not process it?
4. If yes, in what way do you do the processing?

| **Type of product** | **Who does the processing?** | **How do you process it?** | **How much time does it take?** | **How many fruits do you need for it?** | **Purpose of processing** | **For how much money can you sell one unit?** |
| --- | --- | --- | --- | --- | --- | --- |
|  |  |  |  |  |  |  |
|  |  |  |  |  |  |  |
|  |  |  |  |  |  |  |
|  |  |  |  |  |  |  |
|  |  |  |  |  |  |  |

**Codes: Type of product**: 1. Fresh 2. Sun Dry 3. Jam 4. Juice 5. Alcoholic beverage 6. Powder 7.Porridge 8. Oil 9. Nut extraction 10. Other(Specify)

**Purpose**: 1. Easier to store/transport 2. Add value 3. Preservation 4. Other reasons

1. **Storage**
2. Do you store your baobab fruits / processed products?

Yes { } No { }

1. If no, why not?
2. If yes, how and in what form do you store them?

|  | **Baobab Fruit / Product** | **How do you store them (packaging, traditional ways)** | **Location of Storage** | **Shelf Life (Duration)**  **Usual and potential shelf life** | **Losses** | **Reson for losses** | **Solution to reduce losses** | **Did you ever try?** |
| --- | --- | --- | --- | --- | --- | --- | --- | --- |
| 1 |  |  |  |  |  |  |  |  |
| 2 |  |  |  |  |  |  |  |  |
| 3 |  |  |  |  |  |  |  |  |
| 4 |  |  |  |  |  |  |  |  |
| 5 |  |  |  |  |  |  |  |  |
| 6 |  |  |  |  |  |  |  |  |

1. Do you encounter any post-harvest losses during storage?

Yes { } No { }

1. If yes, what nature are the losses, their cause and possible remedies?

|  | **Type of Loss** | **Possible Cause** | **Possible Remedy** |
| --- | --- | --- | --- |
| 1 |  |  |  |
| 2 |  |  |  |
| 3 |  |  |  |
| 4 |  |  |  |
| 5 |  |  |  |
| 6 |  |  |  |
| 7 |  |  |  |

1. **Sales Data**
2. Do you sell your baobab fruits / products

Yes { } No { }

1. If no: Why not? And then continue with number 14)

| **Baobab product** | **When do you sell it?**  **Se** | **Who buys it?** | **Place / Location of Sale**  **1. From home**  **2. At Local**  **Market**  **3. From**  **Major**  **Town**  **Markets**  **4. Export**  **5. Other (Specify)** | **Mode of Transport**  **1. Donkey**  **2. Camel**  **3. Pickup**  **4. Lorry**  **5. Wheel**  **Burrow**  **6. Hand**  **Cart**  **7. Oxen**  **8. Manpower**  **9. Other**  **(Specify)** | **Actual Transport**  **Cost (K.Shs)** | **Do you sort before you sell?**  **Quality**  **1. Very**  **Good**  **2. Good**  **3. Fair**  **4. Poor**  **5. Very**  **Poor** | **Price (K.Shs/kg)**  **Buyer (Who Do You Sell To)**  **1. Local**  **Shopkeepers**  **2. Super markets**  **3. Middlemen**  **4. Local Market**  **Vendors**  **5. Exporters**  **6. Processors**  **7. Other (Specify)** | **Other costs: Sales Tax / Charges**  **(K.Shs)** | **Who sells?** | **Who gets the earning?** |
| --- | --- | --- | --- | --- | --- | --- | --- | --- | --- | --- |
|  |  |  |  |  |  |  |  |  |  |  |
|  |  |  |  |  |  |  |  |  |  |  |
|  |  |  |  |  |  |  |  |  |  |  |
|  |  |  |  |  |  |  |  |  |  |  |
|  |  |  |  |  |  |  |  |  |  |  |
|  |  |  |  |  |  |  |  |  |  |  |
|  |  |  |  |  |  |  |  |  |  |  |
|  |  |  |  |  |  |  |  |  |  |  |
|  |  |  |  |  |  |  |  |  |  |  |
|  |  |  |  |  |  |  |  |  |  |  |
|  |  |  |  |  |  |  |  |  |  |  |
|  |  |  |  |  |  |  |  |  |  |  |
|  |  |  |  |  |  |  |  |  |  |  |

Codes: 1. Father 2. Mother 3. Own 4. Grandparents 5. Employer 6. Other (Specify) ………………………………………………………………………………………..

1. How far is the point of sale for your baobab produce from your farm? …………………km
2. How long does it take to move your produce to the point of sale? ……………….min / hrs
3. What major problems do you encounter in trading with baobab?

| **Problem** | **Rank** | **Possible Solution** |
| --- | --- | --- |
|  |  |  |
|  |  |  |
|  |  |  |
|  |  |  |
|  |  |  |
|  |  |  |

1. **Trade + Quality Requirements**
2. For how long have you been trading in baobab / baobab products? ……………………………………….
3. Who are the most important buyers? Why?
   - 1. ………………………………………………………………………………………….
     2. ………………………………………………………………………………………….
     3. ………………………………………………………………………………………….
4. Have the buyers changed during the last years? If so, how and why: Reasons?
   - 1. ………………………………………………………………………………………………
5. Are there any quality requirements?
6. What are quality requirements imposed by the different baobab buyers?

| **No.** | **Quality requirements** | **Do they pay different prices?** |
| --- | --- | --- |
| 1. |  |  |
| 2. |  |  |
| 3. |  |  |
| 4. |  |  |
| 5. |  |  |
| 6. |  |  |
| 7. |  |  |

1. If you think about the past: Were there trends in change of prices?

Yes { } No { }

If yes: name the trends and give possible reasons

1. If you think about the past: Were there trends in change of volumes?

Yes { } No { }

1. If yes: How would you describe trends in the volume of baobab products? Can you provide us with reasons for the observed trends?
2. If you think in the future- do you expect changes concerning the quantity sold?
3. If you think about the future- do you expect changes concerning the prices for baobab?

|  | **Future trends** | **Possible Reasons** |
| --- | --- | --- |
| **Quantity sold** |  |  |
|  |  |  |
|  |  |  |
| **Price** |  |  |
|  |  |  |
|  |  |  |
|  |  |  |

1=Increased, 2=Decreased, 3=Remained the same, 4=I don’t know

^2^1=Increase, 2=Decrease, 3=Remain the same, 4=I don’t know

1. Do you sell it all in once or in braches/tranches?
2. Do you sell it at peak season or later?
3. What are the reasons for the fluctuation in prices? Is there anything you can do against it?
4. Are there options to sell baobab products collectively? Do you think it is an advantage? If not, reason.
5. **Training**
6. Have you ever received training for planting/marketing/management
7. Has anyone in your family been trained in baobab trading / marketing?

Yes { } No { }

1. If yes, who was trained?
2. Husband 2. Wife 3. Son 4. Daughter 5. Grandchild 6. Employee 7. Other (Specify)……………………………………
3. Who was the training provider? Erst general trees, und dann für baobab

| **No.** | **Training Provider** | **1 – Yes, 2 – No** | **How Often Do They Train You** |
| --- | --- | --- | --- |
| 1. | Research Institutions |  |  |
| 2. | Government Extension Workers |  |  |
| 3. | Private Company |  |  |
| 4. | Farmers Group |  |  |
| 5. | Universities |  |  |
| 6. | Others (Specify) |  |  |

Would you like to have extension? If so : For what?

1. **Baobab Fruit Tree Improvement Strategies**
2. Would you like to have more baobab trees in your farm?

Yes { } No { }

1. If yes, why don’t you have more already?
2. …………………………………………………………………………
3. …………………………………………………………………………
4. …………………………………………………………………………
5. …………………………………………………………………………
6. Are there other bottlenecks concerning storage, marketing, trade- if so name them please
7. Is there anywhere where you could get seedlings from?

Yes { } No { } Where and name ?……………………………………………

1. Please let me know if you agree or disagree with the following statements

[Categories: {1} Strongly Agree {2} Agree {3} Not so Sure {4} Disagree {5} Strongly Disagree]

- 1. Baobab products from my farm do NOT contribute much to the livelihood of me and my family ...............
  2. Baobab fruits from my farm help me and my family to cope with the hunger season ………………..
  3. If I would have enough money, I would not use baobab fruits anymore …….
  4. Baobab fruits are important for the health of my children ………………
  5. If I could, I would like to remove all my baobab trees from my farm ……………..

1. Do you have any other comments and suggestions?

………………………………………………………………………………………………………………………………………………………….

…………………………………………………………………………………………………………………………………………………………

………………………………………………………………………………………………………………………………………………………….

………………………………………………………………………………………………………………………………………………………….

1. **Importance of baobab for your income:**

***To conclude we would like to know how important baobab is for your income.***

1. Which proportion of your total on farm income refers to baobab?
2. Could you give a rough estimate of your baobab income per year?

Ranges:

1. Now include baobab, how much is your on farm income per year in total. Ranges (scoping study)
2. Do you have off-farm employment?
3. Yes { } No { }
4. If yes: In which range lies your total family income per year?

Ranges (scoping study)

*If fit is too complicated: Use seeds to visualize proportions.*

*Thank you very much for your contribution!*

**Methods S3 – Focus Group Discussion**


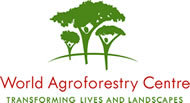


**Focus group discussion no.** **Meeting place**

**Date of FGD** **Name of village/community**

**Supervisor**

**Moderator**

**Translator**

**Welcome**

The World Agro-forestry Centre (ICRAF) is an agricultural research center, headquartered in Nairobi. The Center undertakes research on integrating trees into landscapes for environmental and livelihood benefits.

ICRAF is conducting a pilot study on Baobab Fruit trees in the Taita-Taveta area. We would like to learn from your experiences with baobab. In this Focus Group Discussion I would like to know more about young Baobab trees, and I would like to learn about the distribution of Baobab, where one can find young trees, and whether they grow well. We would also like to know about how you use baobab, how important it is to you, and whether you know any stories about it. Last but not least, I would like to know if there are any differences between how you and your children value and use Baobab.

Your participation is important since it will help in providing crucial knowledge for future benefit in baobab development in Kenya. Please note that your response will be used only by the student Sahrah Fischer and will be treated confidentially.

**List of Participants**

| Name | Gender | Tribe | Contact Number | Village |
| --- | --- | --- | --- | --- |
| 1. |  |  |  |  |
| 2. |  |  |  |  |
| 3. |  |  |  |  |
| 4. |  |  |  |  |
| 5. |  |  |  |  |
| 6. |  |  |  |  |
| 7. |  |  |  |  |
| 8. |  |  |  |  |

**Questions**

1. **Distribution, Management, and Rejuvination**
2. Do Baobab trees grow well in this area?
3. If you think of the past 50 years, has the number of Baobab trees always been the same or has there been a change?
   1. If there has been a change, has the number been higher or lower?
   2. If there has been a change, has this change (higher or lower) been fluctuating or constant?
4. Do you have young Baobabs or seedlings in the area?
   1. Do they survive?
   2. When was the last time you saw a baobab seedling?
   3. When was the last time a baobab seedling grew into an adult tree in this area?
   4. Is there an area where seedlings grow more frequently or better than in other areas (such as rangeland, cropland, boundary, or near homesteads)?
   5. Is a baobab seedling more likely to survive in government land, private land, or common land, or does it not make a difference?
5. Are there Baobabs in this area that have fallen over or died, and what is the main cause of their death (ex. People, Animals, Climate)?
   1. Do Baobabs get cut down? Why?
   2. Do Baobabs get removed as seedlings? Why?
6. What would happen if you would remove an adult Baobab tree? What would happen if you would remove a young baobab tree(seedling)?
   - 1. I heard of a story that if you cut down a baobab tree the elephants get angry and come to your shamba, and that every Baobab tree has its djinni. Do you know these stories? Do you have any other stories? Are these stories from your tribe or from another tribe?
     2. I heard of a law that protects the Baobab tree, is it enforced? Who enforces it? What is the punishment for cutting down a baobab?
     3. Does the same thing happen when you remove a young Baobab tree? Why/Why not?
7. **Occurrences that affect Baobab**
   1. Have there been any events that have changed the number of baobabs in the area? If yes what is the reason? (Please list as many as you can).

*The list should then be written into the drawn grid structure, using either words or pictures, depending on the education and preference of the participants.*

|  | How has Baobab changed? | | | | |
| --- | --- | --- | --- | --- | --- |
| Event/Occurrence | (2)Very decreasing effect / IMAGE | (1) Slightly decreasing effect / IMAGE | (0) Not affected / IMAGE | 1. Slightly increasing effect / IMAGE | 1. Very increasing effect / IMAGE |
| Event 1 |  |  |  |  |  |
| Event 2 |  |  |  |  |  |

*Key:* (2) Very decreasing effect: Baobab death (young and old), either knocked down, fallen, or disease, no seedlings survive

1. Slight decreasing effect: Bad leafing, Bad fruiting (quality and quantity), little growth, few seedling surviving

(0) Not affect meaning that nothing happened and the population stayed the same the entire time

1. Slightly increasing effect: good fruiting (quantity and quality), good leafing, seedlings grow and few survive
2. Very increasing effect: All seedlings survive and grow, excellent fruiting, noticeable growth

***Note to Moderator:*** *The events mentioned will be discussed in an interactive group activity*, *to identify any other factors that would also influence the effect the event in question has on Baobabs. An example would be the location of the Baobabs (such as whether they grow in Bushland or in Cropland) or the age of Baobab (for example if there is a pest, and younger Baobabs are more affected than the older Baobabs).*

- ***Linked to 2.a)*** *To fill out the grid, each participant will receive at least one pebble. If separate factors are identified, then each participant will receive pebbles of different colours. For example, if the location of the Baobab is a factor, then each participant will receive two pebbles in different colours. One colour pebble will represent the Baobabs in the Bushland and a pebble of a different colour will represent the Baobabs growing in the Cropland.*
- ***Linked to 2.b)****The participants will then decide the effect (impact of the change) the event has on Baobab, and place their pebbles into the grid. To come to a final decision the participants can discuss their decisions and move the pebbles around the grid. If there are more factors, then the entire process will be repeated until all factors for the event are covered. Once all factors for the event have been rated, we will move on to the next event.*
  1. *The following lists contains a list of events.* ***If these events have not been mentioned above****, I would like the participants to also discuss these, to see whether they have any effect on Baobab.*

I would like to know a little bit more about these events. Do you know if they have any effect on the Baobab population, like the events that you listed before?

- - 1. Land use – was any part of this land used differently before? (Ex. Was part of this land community land and is now government land?)
    2. Drought events (Mwatate timeline) – drought since 2007
    3. Wildlife management by KWS, such as the construction of the fence
    4. Increase of Livestock density
    5. Increase of population density
    6. Land consolidation
    7. Pests

1. **Perception**
   1. Do you use Baobab at home?
      1. Where do you get it from?
      2. What do you use it for?
      3. Do you have it all year round or only in a specific time?
      4. Do you sell it?
   2. Who eats Baobab?
      1. What would you think of a man/woman/child sitting by the side of the road eating baobab?
      2. Is there anybody that shouldn’t eat Baobab, and why? Is there anybody that should eat Baobab and why?
      3. Is there any time when one should eat baobab? Is there a time when one should avoid eating baobab?
   3. IN AREAS WITH MANY TREES: Is Baobab important to you? If someone would come and offer to remove all of your baobabs for free, what would you say?

IN AREAS WITH FEW TREES: If someone would come and offer to plant many baobabs for you, what would you say?

- 1. How do young people see Baobab? Are there any differences to the way that you value it? What are they?
